# Magnesium as a Bioenergetic Checkpoint Linking Mitochondrial Function, Metabolic Disease, and Aging

**DOI:** 10.1111/acel.70578

**Published:** 2026-06-04

**Authors:** Chien‐Wei Huang, Chen‐Yueh Wen, Andy P. Tsai, Boyang Wang, Kuan‐Hao Tsui, Yu‐Juei Hsu, Chia‐Jung Li

**Affiliations:** ^1^ Division of Nephrology, Department of Internal Medicine Kaohsiung Veterans General Hospital Kaohsiung Taiwan; ^2^ School of Medicine National Yang Ming Chiao Tung University Taipei Taiwan; ^3^ Division of Urology Show Chwan Memorial Hospital Changhua Taiwan; ^4^ Department of Neurology and Neurological Sciences Stanford University School of Medicine Stanford California USA; ^5^ School of Computing National University of Singapore Singapore Singapore; ^6^ Department of Obstetrics and Gynecology Kaohsiung Veterans General Hospital Kaohsiung Taiwan; ^7^ Institute of Biopharmaceutical Sciences National Sun Yat‐Sen University Kaohsiung Taiwan; ^8^ Division of Nephrology, Department of Medicine Tri‐Service General Hospital, National Defense Medical University Taipei Taiwan; ^9^ Graduate Institute of Biochemistry, College of Biomedical Sciences National Defense Medical University Taipei Taiwan; ^10^ Institute of Cellular and System Medicine National Health Research Institutes Zhunan Taiwan; ^11^ National Museum of Marine Biology & Aquarium Checheng Pingtung Taiwan

**Keywords:** aging and senescence, kidney injury, magnesium, metabolic disease, mitochondria

## Abstract

Magnesium is traditionally viewed as a permissive electrolyte required for cellular viability. Emerging evidence, however, reveals a more central role for Mg^2+^ as an active regulator of mitochondrial bioenergetics and metabolic resilience. In this Review, we synthesize recent advances in renal magnesium handling, mitochondrial Mg^2+^ transport, and MgATP chemistry to propose a unifying framework in which magnesium functions as a bioenergetic checkpoint. At the cellular level, Mg^2+^ availability specifies the functional pool of ATP, constrains kinase signaling, and stabilizes mitochondrial performance by limiting calcium overload and oxidative stress. At the tissue and organismal levels, disruption of magnesium homeostasis contributes to metabolic inflexibility, insulin resistance, acute kidney injury, and the progressive decline in stress tolerance that accompanies aging. We further discuss how age‐associated drift in mitochondrial magnesium may act as a hidden temporal regulator that lowers the threshold for cellular senescence. Finally, we outline emerging therapeutic strategies, including transport‐informed and compartment‐specific approaches, that move beyond nonspecific supplementation toward precision modulation of magnesium‐dependent bioenergetics. Together, this framework positions magnesium as a mechanistic link between mitochondrial function, metabolic disease, and aging, with broad implications for translational intervention.

## Graphical abstract

This review positions magnesium as a central bioenergetic checkpoint that orchestrates mitochondrial function and metabolic resilience. By defining the functional ATP pool and stabilizing mitochondrial performance, magnesium homeostasis serves as a mechanistic link between renal handling, metabolic disease, and the aging process.

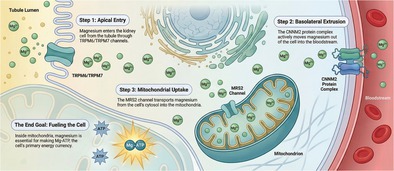

## 1 Introduction

Although the kidney accounts for less than 1% of total body mass, it consumes 20%–25% of resting oxygen. This metabolic rate is comparable only to the heart (Bhargava and Schnellmann 2017; O'Connor 2006). This extraordinary bioenergetic demand is not an optional feature but a physiological mandate driven by the relentless requirement for solute transport. Every day, the human kidneys filter approximately 180 L of plasma, recovering 99% of water and electrolytes to maintain systemic homeostasis (Weiner et al. 2015). This thermodynamic feat relies almost exclusively on the active transport machinery of the proximal tubular epithelial cells (PTECs), which contain a high density of mitochondria to power the Na^+^/K^+^/ATPase pumps (Soltoff 1986). Consequently, the kidney operates on the precipice of hypoxia, with PTECs functioning as obligate aerobes that are exquisitely sensitive to perturbations in oxygen and nutrient supply (Osada et al. 2025). In the context of acute kidney injury (AKI) and its progression to chronic kidney disease (CKD), this energetic fragility becomes the central driver of pathology. It is now well‐established that the failure of renal repair is fundamentally a metabolic failure (Tran et al. 2011). Following ischemic or toxic insults, surviving tubular cells undergo a profound metabolic reprogramming, characterized by the suppression of mitochondrial fatty acid oxidation (FAO) and a compensatory shift toward aerobic glycolysis (Kang et al. 2015). While this “Warburg‐like” effect initially serves as an adaptive survival mechanism to maintain ATP production under hypoxic conditions, its persistence is maladaptive (Faubert et al. 2020). The sustained decoupling of glycolysis from oxidative phosphorylation (OXPHOS) deprives the kidney of its primary high‐efficiency energy source, leading to ATP depletion, lipid accumulation (lipotoxicity), and the induction of a pro‐fibrotic senescence‐associated secretory phenotype (SASP; Christov et al. 2013; Miguel et al. 2021).

However, current models of renal bioenergetics focus on carbon substrates such as fatty acids, glucose, and glutamine, and remain incomplete. By fixating on the “fuel,” we have largely overlooked the “machinery” that converts this fuel into work, specifically the ionic microenvironment that dictates mitochondrial efficiency (Anselme et al. 2025). ATP does not exist in isolation within the cellular milieu; it is biologically active only as a magnesium‐ATP complex (Gupta and Moore 1980). Magnesium, the second most abundant intracellular cation, is an essential cofactor for over 600 enzymatic reactions, including every step of glycolysis and the TCA cycle, as well as the catalytic activity of ATP synthase itself (de Baaij et al. 2015). Therefore, a deficiency in intracellular magnesium constitutes a fundamental “energetic brake,” rendering the available ATP pool functionally inert and stalling metabolic flux regardless of substrate availability. More critically, emerging evidence suggests that magnesium functions as a signaling “gatekeeper” for mitochondrial integrity, acting in direct antagonism to calcium (Ca^2+^; Pilchova et al. 2017). Under physiological conditions, cytosolic magnesium inhibits the mitochondrial calcium uniporter (MCU), limiting calcium influx into the mitochondrial matrix (Kirichok et al. 2004). In states of renal injury, particularly those induced by nephrotoxins like cisplatin or ischemia–reperfusion, the rapid loss of intracellular magnesium disinhibits the MCU, leading to catastrophic mitochondrial calcium overload (Liu, Wang, Qiao, et al. 2025). This calcium surge triggers the opening of the mitochondrial permeability transition pore (mPTP), collapses the transmembrane potential (ΔΨm), and uncouples respiration, thereby converting the mitochondrion from an energy generator into a generator of reactive oxygen species (ROS) and a mediator of regulated cell death (necroptosis and ferroptosis; Bernardi and Di Lisa 2015; Linkermann et al. 2014).

**FIGURE 1:**
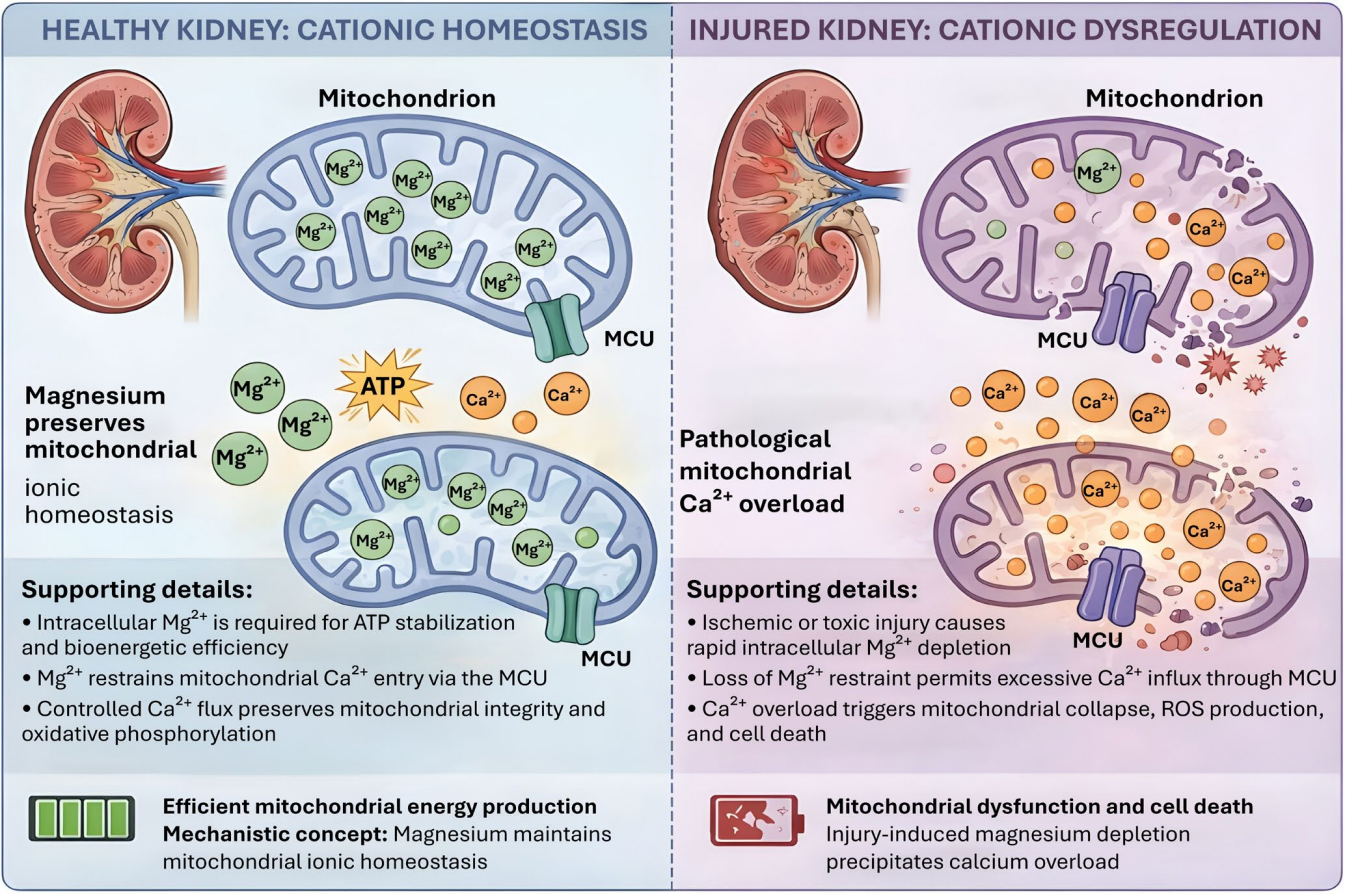
Magnesium maintains mitochondrial cationic balance and prevents bioenergetic collapse in the kidney. Left, under physiological conditions, intracellular Mg2+ is maintained at sufficient levels to support Mg–ATP formation and to restrain mitochondrial Ca^2+^ entry through the mitochondrial calcium uniporter (MCU). Adequate matrix Mg^2+^ acts as a functional “mitochondrial guardian,” ensuring efficient oxidative phosphorylation, controlled Ca^2+^ signaling, and sustained ATP production required for renal tubular function. Right, during kidney injury, insults such as ischemia, hypoxia, or nephrotoxic stress trigger rapid intracellular Mg^2+^ depletion. Loss of Mg^2+^ removes this permissive constraint on Ca^2+^ influx, leading to unchecked mitochondrial Ca^2+^ overload, opening of permeability transition pathways, collapse of mitochondrial membrane potential, and failure of ATP synthesis. This cationic collapse promotes excessive reactive oxygen species (ROS) generation, mitochondrial dysfunction, and ultimately cell death and fibrotic remodeling. Together, the schematic illustrates how Mg^2+^ availability defines a bioenergetic checkpoint that separates adaptive mitochondrial activation from catastrophic failure in renal epithelial cells.

Despite these mechanistic insights, magnesium homeostasis is frequently relegated to a bystander role in renal pathology, viewed merely as a biomarker of tubular damage rather than a driver of it. This oversight is significant given that the kidney is the master regulator of systemic magnesium balance, with the distal convoluted tubule (DCT) fine‐tuning reabsorption via the TRPM6 channel (Schlingmann et al. 2002). The “AKI‐to‐CKD transition” may thus represent not just a failure of FAO, but a collapse of the “Mg‐Ca‐Mitochondria Axis,” where the loss of magnesium buffering capacity locks the tubule in a state of metabolic rigidity and calcium toxicity (Pham et al. 2014). In this Review, we synthesize the evidence linking cationic dysregulation to metabolic failure in kidney disease (Figure 1). We move beyond the traditional view of magnesium as a simple electrolyte to propose a new conceptual model: that mitochondrial magnesium homeostasis serves as a critical metabolic checkpoint. We will first outline the molecular machinery of renal magnesium handling, then dissect the biophysical antagonism between magnesium and calcium at the mitochondrial membrane, and finally explore how restoring this cationic balance could unlock new therapeutic avenues to halt the metabolic catastrophe driving renal fibrosis (Ponnusamy et al. 2024).

## 2 Molecular Machinery of Magnesium Spatial Handling: from Nephron to Mitochondria

In renal epithelia, magnesium handling is organized as a directional relay that links trans‐epithelial flux to intracellular bioenergetics. Apical entry is primarily governed by the TRPM6/TRPM7 chanzyme complex, whereas basolateral extrusion is tuned by the CNNM2 regulatory module and its interacting partners. Once in the cytosol, Mg^2+^ is not simply buffered but actively routed to mitochondria, where matrix Mg^2+^ availability is constrained by inner‐membrane transport and membrane potential. In this section, we map these layers from nephron‐facing transport to mitochondrial uptake and show how they converge on a shared functional endpoint: the maintenance of Mg‐dependent metabolic competence.

### 2.1 TRPM6/TRPM7 Chanzyme Complex as the Gatekeeper of Renal Magnesium Reabsorption

**FIGURE 2:**
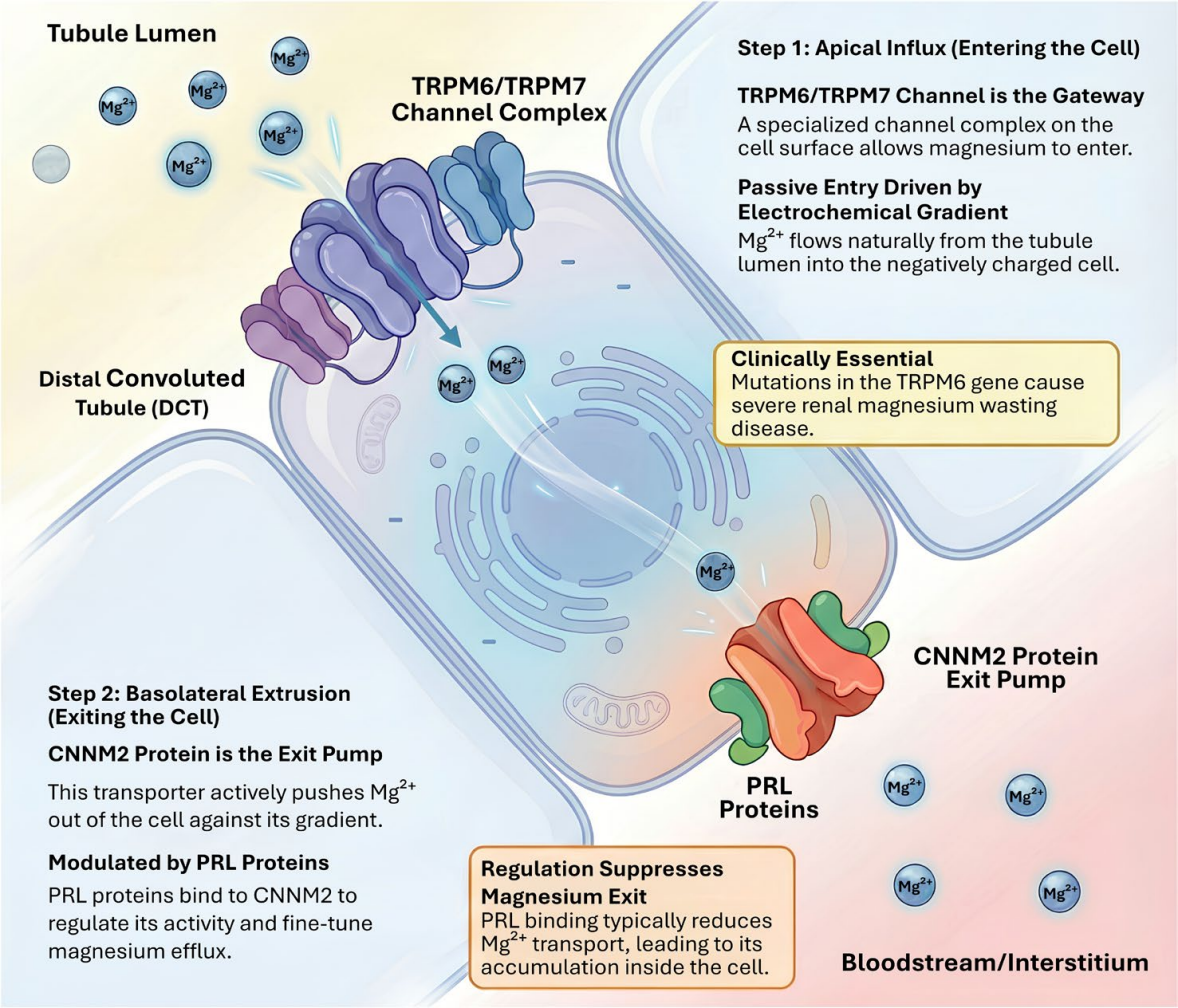
Directional magnesium transport in the distal nephron establishes intracellular magnesium availability. Magnesium reabsorption in renal distal convoluted tubule cells is organized as a directional, two‐step process. Step 1 (apical influx): Mg^2+^ enters epithelial cells from the tubular lumen through the TRPM6/TRPM7 channel complex, driven primarily by the electrochemical gradient across the apical membrane. This pathway represents the principal gateway for transcellular magnesium uptake, and loss‐of‐function mutations in TRPM6 cause severe renal magnesium wasting. Step 2 (basolateral extrusion): Cytosolic Mg^2+^ is exported into the interstitium and bloodstream by the CNNM2 protein complex at the basolateral membrane. CNNM2‐mediated magnesium efflux is dynamically regulated by phosphatases of regenerating liver (PRL) proteins, which bind to CNNM2 and suppress magnesium exit, thereby fine‐tuning intracellular magnesium retention. Together, this schematic illustrates how coordinated apical entry and regulated basolateral extrusion determine intracellular Mg^2+^ availability, providing the upstream foundation for magnesium‐dependent metabolic and mitochondrial functions.

Systemic magnesium balance relies on a dynamic equilibrium between intestinal absorption, renal excretion, and skeletal storage. While the glomerulus filters a substantial load of magnesium daily, and the thick ascending limb (TAL) reabsorbs the majority via passive paracellular routes (de Baaij et al. 2015; Khan and Khan 2025), the definitive fine‐tuning of urinary excretion occurs exclusively in the distal convoluted tubule (DCT). Here, transport is active, transcellular, and dependent on a highly specialized apical entry system. The primary molecular entities mediating this influx are the Transient Receptor Potential Melastatin (TRPM) family members, specifically TRPM6 and TRPM7 (Dimke et al. 2011). While TRPM7 is ubiquitously expressed and essential for cell viability, TRPM6 expression is strictly confined to the apical membranes of the DCT and intestinal epithelia (Mittermeier et al. 2019; Vargas‐Poussou et al. 2023). Cryo‐electron microscopy (cryo‐EM) studies have resolved that, under physiological conditions in the DCT, the functional apical channel assembles predominantly as a heterotetrameric complex comprising both TRPM6 and TRPM7 subunits (Chubanov et al. 2016; Schmidt et al. 2022). These proteins exhibit a unique chanzyme architecture that represents an evolutionary fusion of an N terminal ion channel pore with a C terminal alpha kinase domain. The channel pore displays high selectivity for divalent cations, with magnesium favored over calcium, and functions broadly as a constitutively active channel (Zhang et al. 2023). The driving force for this apical entry is strictly electrochemical; the intracellular potential of the DCT cell is maintained at a negative value relative to the tubular lumen, creating a gradient that favors the passive entry of Mg^2+^ through the open pore (Voets et al. 2004; Figure 2). The clinical indispensability of this specific machinery is evidenced by Hypomagnesemia with Secondary Hypocalcemia (HSH), where loss‐of‐function mutations in TRPM6 cause severe renal magnesium wasting that cannot be compensated by TRPM7 homomers alone (Schlingmann et al. 2002; Walder et al. 2002).

### 2.2 The CNNM2–PRL Module and the Regulation of Basolateral Magnesium Extrusion

Following apical entry, intracellular Mg^2+^ must be extruded across the basolateral membrane into the renal interstitium against both chemical and electrical gradients. This thermodynamically unfavorable step constitutes the rate‐limiting barrier in transepithelial magnesium transport. The molecular identity of this extrusion system has been confirmed as the Cyclin M (CNNM) family of proteins, specifically CNNM2 (Funato and Miki 2022). Genetic studies have causally linked *CNNM2* mutations to dominant familial hypomagnesemia, confirming its non‐redundant role in renal magnesium retention (Liu, Wang, Wang, et al. 2025). Structurally, CNNM2 contains a transmembrane DUF21 domain and a cytosolic cystathionine‐β‐synthase (CBS) pair domain (Gimenez‐Mascarell et al. 2019). While the precise stoichiometry of transport, specifically whether CNNM2 functions as a sodium magnesium exchanger that exploits the inward sodium gradient generated by the sodium potassium ATPase, remains under active biophysical investigation, the requirement of CNNM2 for magnesium efflux is well established (Yamazaki et al. 2013; Figure 2). A critical regulatory layer at this interface involves the phosphatases of regenerating liver (PRLs), which directly engage the CBS pair module of CNNM transporters (Gimenez‐Mascarell et al. 2017; Gulerez et al. 2016). Biochemical analyses indicate that dimerization of the CBS pair in solution is tightly coupled to Mg–ATP binding and can be further enhanced by PRL association, consistent with PRLs reshaping, rather than abolishing, the nucleotide‐dependent conformational landscape of CNNMs (Chen et al. 2020). Functionally, PRL binding has been most consistently linked to suppression of CNNM‐mediated Mg^2+^ efflux and consequent intracellular Mg^2+^ accumulation, positioning PRLs as modulators that tune epithelial magnesium handling capacity rather than as constitutive activators of export (Gulerez et al. 2016).

### 2.3 The Mitochondrial Interface: Balancing Magnesium Sequestration and Membrane Potential

**FIGURE 3:**
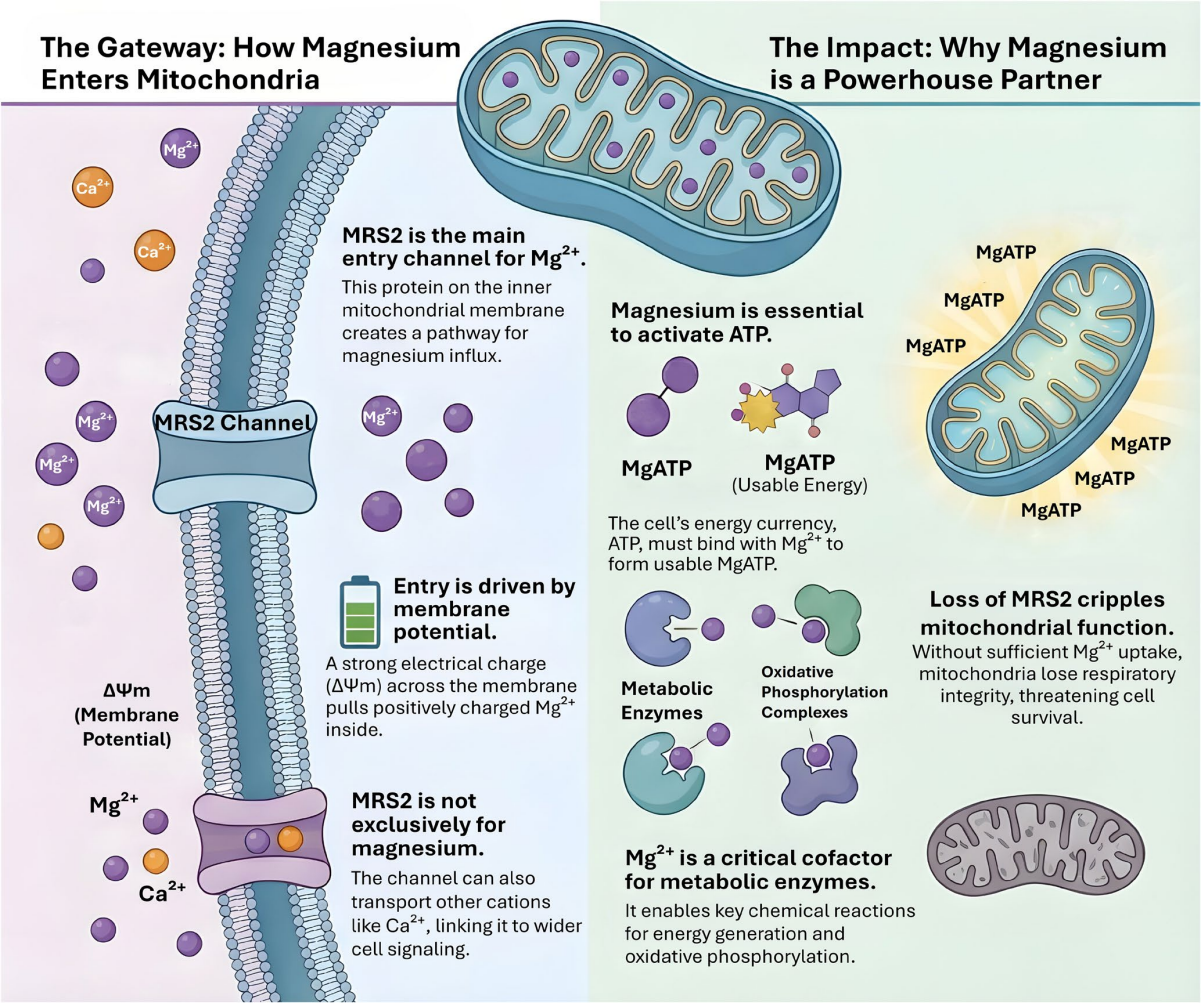
Mitochondrial magnesium uptake via MRS2 couples membrane potential to bioenergetic capacity. Magnesium entry into the mitochondrial matrix is primarily mediated by the inner‐membrane channel MRS2. Driven by the mitochondrial membrane potential (ΔΨm), positively charged Mg^2+^ is electrophoretically transported from the cytosol into the matrix, coupling magnesium uptake directly to mitochondrial bioenergetic status. Within mitochondria, Mg^2+^ is required to activate ATP, forming MgATP as the bioactive energy currency, and serves as an essential cofactor for metabolic enzymes and oxidative phosphorylation complexes. Adequate mitochondrial Mg^2+^ availability thereby sustains respiratory integrity and ATP production. Conversely, the loss or dysfunction of MRS2 limits magnesium uptake, disrupts MgATP chemistry, and compromises mitochondrial function, ultimately threatening cellular energy homeostasis and survival. Together, this schematic illustrates how MRS2 positions magnesium as a key integrator of membrane potential, enzymatic activity, and mitochondrial performance.

Intracellular magnesium homeostasis is tightly compartmentalized, with a substantial fraction of total cellular Mg^2+^ sequestered in organelles where it is largely buffered by phospholipids, nucleotides, and proteins. Within this landscape, mitochondria act as a major, dynamically exchangeable Mg^2+^ pool that couples cation availability to bioenergetic demand (Yamanaka et al. 2016). Importantly, quantitative statements must distinguish between total Mg (dominated by the bound pool) and free Mg^2+^ (the signaling and catalytic pool). Accordingly, comparisons of mitochondrial Mg content with cytosolic Mg^2+^ should be framed as differences in buffering capacity and exchange dynamics, rather than as a simple free concentration gradient (Yamanaka et al. 2016). The best characterized entry route for Mg^2+^ across the inner mitochondrial membrane is Mitochondrial RNA Splicing 2 (MRS2), a eukaryotic homolog of the bacterial CorA family (Li et al. 2023). Recent cryo‐EM studies converge on a homo‐pentameric architecture that defines a central ion conduction pathway and a gated permeation mechanism, providing a structural basis for Mg^2+^ translocation through the inner membrane (Lai, Balaraman, et al. 2023; Li et al. 2023). Notably, electrophysiological analyses of human MRS2 indicate that channel activity is regulated by Ca^2+^ and that the pore can conduct multiple cations, including Mg^2+^, rather than operating as a strictly Mg^2+^‐selective conduit under all conditions (He et al. 2025). This revised view is mechanistically important, because it places MRS2 at the intersection of mitochondrial cation flux, signaling context, and membrane energization (He et al. 2025; Li et al. 2023). In energetic terms, net cation influx through an inner‐membrane channel is ultimately shaped by the ΔΨm, which provides the electrical component of the driving force for positively charged ions. Therefore, when ΔΨm collapses, the electrical incentive for mitochondrial Mg^2+^ accumulation diminishes, functionally uncoupling Mg^2+^ uptake from oxidative metabolism (Yamanaka et al. 2016). In this sense, MRS2‐mediated Mg^2+^ entry is best described as channel permeation that is gated by channel state and constrained by the prevailing electrochemical landscape, rather than as an ATP‐driven pumping process (He et al. 2025; Li et al. 2023; Madaris et al. 2023). As illustrated in Figure 3, mitochondrial magnesium uptake through MRS2 directly couples membrane potential to functional energy availability.

### 2.4 The Magnesium–Bioenergetic Bridge for Metabolic Coupling

The physiological imperative for mitochondrial Mg^2+^ uptake lies in how Mg integrates catalysis, nucleotide chemistry, and respiratory performance. Magnesium is an obligate cofactor for numerous metabolic enzymes, and in mitochondria it supports reaction chemistry across central carbon metabolism and oxidative phosphorylation (Yamanaka et al. 2016). A particularly consequential constraint is that ATP is predominantly used in cells as a MgATP complex, meaning that “ATP sufficiency” is inseparable from Mg availability at the site of ATP synthesis and utilization (Yamanaka et al. 2016). Thus, mitochondrial Mg handling is not a passive bookkeeping exercise in ion balance, but a prerequisite for sustaining the chemical currency that powers phosphorylation networks and transport work (Yamanaka et al. 2016). Consistent with this logic, genetic or functional impairment of MRS2 compromises mitochondrial Mg homeostasis and disrupts mitochondrial metabolism. In cellular systems, MRS2 depletion reduces mitochondrial Mg uptake and undermines bioenergetic resilience under stress (Yamanaka et al. 2016). In vivo, inducible loss of MRS2 has been linked to profound mitochondrial dysfunction, including loss of respiratory chain integrity with severe consequences for cell viability (Li et al. 2023). Together with the recent structural and functional dissection of human MRS2, these findings support the concept that MRS2 is a necessary determinant of mitochondrial Mg homeostasis that helps preserve oxidative phosphorylation capacity in demanding contexts (Madaris et al. 2023; Mastrototaro et al. 2016; Figure 3). Finally, Mg^2+^ flux is bidirectional at the organelle level. A mitochondrial Mg^2+^ efflux pathway has been attributed to SLC41A3, providing a mechanism to extrude Mg^2+^ and thereby prevent pathological accumulation. In aggregate, current evidence positions MRS2 as the core, best established molecular entity required for mitochondrial Mg^2+^ influx, with efflux mechanisms such as SLC41A3 shaping steady‐state set points and adaptive dynamics (He et al. 2025; Madaris et al. 2023). Collectively, these transport layers define how much Mg^2+^ is available to form Mg–ATP and to support Mg‐dependent enzymatic reactions. TRPM6/TRPM7 sets the entry capacity, CNNM2‐centered regulation determines the efficiency of basolateral export, and MRS2 couples cytosolic supply to matrix sufficiency under bioenergetic constraints. This architecture provides a mechanistic bridge to the next section, where we examine how Mg–ATP coupling and Mg‐sensitive enzyme kinetics translate ion handling into metabolic regulation and disease vulnerability.

## 3 The Bioenergetic Core of MgATP Coupling and Mitochondrial Metabolic Resilience

**FIGURE 4:**
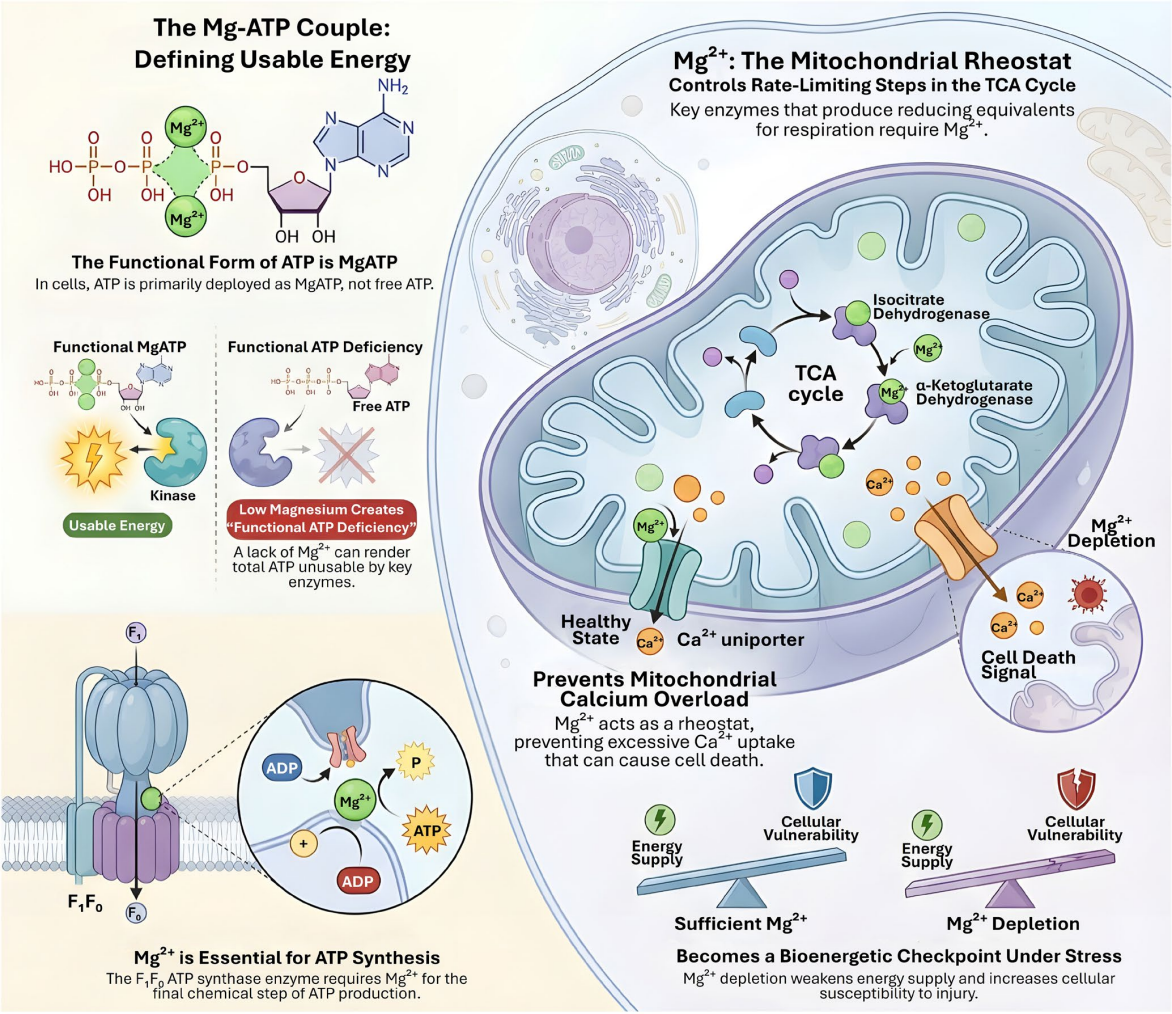
Magnesium defines usable cellular energy and functions as a mitochondrial bioenergetic checkpoint. ATP is biologically active primarily as MgATP rather than free ATP, making Mg^2+^ essential for ATP‐dependent kinase activity and energy utilization. Magnesium deficiency therefore induces a state of “functional ATP deficiency,” in which total ATP may be preserved but cannot be efficiently deployed. Within mitochondria, Mg^2+^ regulates rate‐limiting steps of the tricarboxylic acid cycle, including isocitrate dehydrogenase and α‐ketoglutarate dehydrogenase, and is required for F₁F₀‐ATP synthase activity during ATP production. In addition, mitochondrial Mg^2+^ restrains calcium uptake, limiting Ca^2+^ overload and cell death signaling. Loss of Mg^2+^ disrupts these coupled processes, weakening energy supply and increasing cellular vulnerability to stress, thereby positioning mitochondrial magnesium as a central bioenergetic checkpoint.

Magnesium is often treated as a permissive electrolyte, yet in bioenergetics it functions as a defining cofactor. In cells, ATP is predominantly deployed as MgATP rather than as free ATP. This coordination reshapes phosphate charge and geometry, setting the kinetic feasibility of phosphoryl transfer reactions across kinases and ATPases and thereby determining the functional availability of cellular energy (Daw et al. 2020; He et al. 2025; Madaris et al. 2023). Consequently, Mg^2+^ is not merely upstream of metabolism through its transport machinery, but embedded within the energetic currency that powers metabolic regulation. As illustrated in Figure 4, magnesium availability defines functional ATP usage and operates as a mitochondrial bioenergetic checkpoint under metabolic stress.

### 3.1 Magnesium as a Determinant of Functional ATP Availability

A central implication of MgATP predominance is that fluctuations in free Mg^2+^ can uncouple total ATP abundance from ATP usability. When Mg^2+^ becomes limiting, the pool of MgATP contracts even if adenylate charge appears preserved, creating a state that can be conceptualized as functional ATP deficiency. This is particularly relevant for kinase networks, where the substrate is MgATP rather than ATP per se. Mechanistic enzymology has long established that many kinases coordinate Mg^2+^ in the active site to position the phosphates for in‐line transfer and to stabilize negative charge in the transition state (Gupta and Yushok 1980; Kleczkowski and Igamberdiev 2023). What is newly appreciated is how this chemistry scales into systems level regulation: Mg^2+^ can tune the balance between Mg complexed and Mg free nucleotide species, shifting reaction directionality and apparent affinities across nucleotide interconversion hubs such as adenylate kinase and nucleoside monophosphate kinases (Kleczkowski and Igamberdiev 2025; Nam et al. 2024; Run et al. 2015). A recent structural and enzymatic dissection of adenylate kinase shows that Mg^2+^ is not only an electrostatic cofactor but can induce a substrate reorganization that optimizes the phosphoryl transfer geometry, highlighting how Mg^2+^ can gate adenylate equilibration with direct kinetic consequences (Nam et al. 2024). Because adenylate kinase sets the interconversion between ATP, ADP, and AMP, Mg^2+^ at this node is positioned to influence both energetic buffering and downstream AMP sensitive signaling.

### 3.2 Mitochondrial Magnesium Dynamics in the Coupling of Respiration and Nucleotide Chemistry

Within mitochondria, Mg^2+^ dependence intensifies because oxidative phosphorylation is simultaneously a generator and consumer of Mg coordinated nucleotides. MRS2 mediated Mg^2+^ uptake is increasingly viewed as a determinant of mitochondrial “cationic set point” that shapes bioenergetic resilience (He et al. 2025). Elegant work linking mitochondrial Mg^2+^ to Ca^2+^ handling further supports the idea that matrix Mg^2+^ is a rheostat for the mitochondrial Ca^2+^ uniporter, with reduced matrix Mg^2+^ associating with increased Ca^2+^ uptake and enhanced susceptibility to permeability transition (Ponnusamy et al. 2024; Uthayabalan et al. 2023; Yamanaka et al. 2016). These findings are conceptually important for bioenergetics because Ca^2+^ stimulated dehydrogenase activation can be beneficial, yet Ca^2+^ excess collapses membrane potential and ATP synthesis. Mg^2+^ therefore sits at the intersection of nucleotide chemistry and ion governed control of respiration, enforcing a permissive window in which mitochondrial activation does not tip into catastrophic overload. A striking illustration of this coupling comes from cell level Mg^2+^ dynamics triggered by lactate. In a study that repositioned lactate as a second messenger, L lactate promoted Mg^2+^ release from endoplasmic reticulum stores and subsequent mitochondrial Mg^2+^ uptake, in a process that depended on MRS2 and integrated glycolytic output with mitochondrial function (Daw et al. 2020; Wilde and Christofk 2020). This work reframes Mg^2+^ as a mobile signal that communicates carbon flux state to the mitochondrial matrix, rather than a static cofactor passively distributed by diffusion. It also implies that metabolic states commonly encountered in kidney physiology, including high glycolytic throughput in stressed epithelia, may dynamically reshape matrix Mg^2+^ and thus MgATP dependent enzymology.

### 3.3 Magnesium as an Integral Component of TCA Cycle Flux and Regulation

The tricarboxylic acid cycle contains multiple enzymes that require divalent cations for catalysis and allosteric control, positioning Mg^2+^ as a determinant of flux rather than a background ion. Human NAD dependent isocitrate dehydrogenase (IDH3), which catalyzes oxidative decarboxylation of isocitrate to alpha ketoglutarate, has now been structurally resolved with detailed insights into assembly and allosteric regulation (Sun et al. 2020). These structures and accompanying kinetic analyses clarify how ligand binding and conformational coupling control active site function, and they reinforce that metal ion coordination is a core component of catalytic competence. Alpha ketoglutarate dehydrogenase (OGDH) is another major control point that connects carbon flux to NADH production. cryo EM of the human complex has advanced mechanistic understanding of its activity and regulation, providing a platform for mapping how cofactor occupancy and conformational states influence rate limitation in the cycle (Zhong et al. 2022). While these studies do not reduce regulation to Mg^2+^ alone, they underscore that cation coordinated chemistry is embedded in the key mitochondrial steps that produce reducing equivalents for the respiratory chain.

### 3.4 Mg‐Coordinated Catalysis and Transport in Oxidative Phosphorylation

At the endpoint of mitochondrial energy conversion, the F_1_F_o_ ATP synthase is often conceptualized as a proton driven machine, but its chemistry remains Mg^2+^ centric. High resolution structural work has provided snapshots of the catalytic cycle and conformational transitions, making explicit that nucleotide binding and release events occur within a Mg coordinated framework (Guo and Rubinstein 2022; Lai, Zhang, et al. 2023; Sobti et al. 2021). A complete rotational catalytic cycle of the F1 sector resolved by cryo EM captured ordered state transitions that clarify how nucleotide occupancy couples to mechanical steps. Complementary work examining ATP synthase under strain during catalysis reinforced that conformational energy landscapes, nucleotide binding states, and mechanical deformation are inseparable in the functioning enzyme (Guo and Rubinstein 2022). Human ATP synthase structures across multiple rotational states extend these principles to human mitochondria, providing a critical reference for disease relevant interpretation of OXPHOS dysfunction (Lai, Zhang, et al. 2023). Together, these studies support a mechanistic view in which Mg^2+^ availability can influence not only the chemical step of phosphoryl transfer but also the kinetics of nucleotide exchange, potentially sensitizing ATP production to Mg^2+^ limitation even before gross depolarization occurs. Once synthesized, ATP must be distributed and buffered between compartments. Here, Mg^2+^ dependence reappears in transport. The mitochondrial ATP Mg phosphate carrier SCaMC (SLC25A23 and related paralogs) transports MgATP. This distinguishes it from the standard ADP ATP carrier, which exchanges free nucleotides. Structural and functional analyses have defined an internal binding site that explains MgATP selectivity and identified residues essential for preference toward the Mg complex (Run et al. 2015). This selectivity is not an arcane biophysical feature; it is a design principle that enables mitochondria to traffic Mg coordinated energy currency in contexts where MgATP, rather than free ATP, is the biologically relevant species. In excitable cells, SCaMC family function has been linked to maintenance of mitochondrial ATP under stress, supporting the idea that MgATP transport can shape vulnerability to energetic collapse (Traba et al. 2012). In metabolically demanding epithelia such as kidney tubules, this axis may be particularly relevant because sustained ATP turnover requires not only synthesis but also proper chemical speciation and delivery of ATP.

### 3.5 Magnesium as a Critical Bioenergetic Checkpoint Under Metabolic Stress

A key translational insight is that Mg^2+^ regulation becomes most visible under stress, when homeostatic slack is lost. Limiting MRS2 dependent mitochondrial Mg^2+^ uptake has been shown to induce metabolic programming in the setting of prolonged dietary stress, indicating that mitochondrial Mg^2+^ influx is not redundant but instructive for metabolic remodeling (Madaris et al. 2023). More broadly, perturbations in mitochondrial Mg^2+^ handling can precipitate complex I dysfunction, reduce ATP output, and amplify susceptibility to injury, positioning Mg^2+^ not as an optional micronutrient but as a limiting factor for mitochondrial competence (Ponnusamy et al. 2024; Yamanaka et al. 2016). The emerging picture is that Mg^2+^ availability and compartmentalization define the operating range of oxidative metabolism by controlling MgATP abundance, the kinetics of phosphoryl transfer, and the stability of coupled respiration. This reframing has direct implications for kidney pathophysiology. Renal tubular cells operate near maximal energetic demand, with limited tolerance for inefficiency. In such a context, Mg^2+^ depletion can simultaneously weaken dehydrogenase driven NADH supply, slow ATP synthase chemistry through altered Mg nucleotide occupancy, and destabilize ion homeostasis through enhanced mitochondrial Ca^2+^ uptake and permeability transition susceptibility. These mechanisms provide a coherent bridge between Mg^2+^ handling proteins and downstream injury phenotypes, setting the stage for the Mg Ca mitochondria axis and its role in acute bioenergetic failure.

## 4 Pathophysiology of Magnesium Deficiency: Insulin Resistance and Metabolic Syndrome

Intracellular magnesium deficiency is more than a biomarker of cardiometabolic risk; it can actively erode signaling fidelity, metabolic flux, and stress tolerance in insulin‐responsive tissues. This pathological state is a direct functional extension of the transport and bioenergetic principles established in earlier sections, where the disruption of the TRPM6/CNNM2‐regulated magnesium set point and the resulting functional ATP deficiency manifest as systemic metabolic failure. Hypomagnesemia is common in type 2 diabetes, with pooled observational estimates suggesting a prevalence of roughly one third, albeit with substantial heterogeneity (Pitliya et al. 2024). Mechanistically, Mg^2+^ serves as both cofactor and constraint: when cytosolic free Mg^2+^ declines, insulin signaling becomes less reliable because phosphorylation reactions depend on MgATP, while oxidative and inflammatory stress can further promote renal and cellular Mg^2+^ loss, creating a feed‐forward cycle (Barbagallo and Dominguez 2007; Liamis et al. 2014; Oost et al. 2023).

**FIGURE 5:**
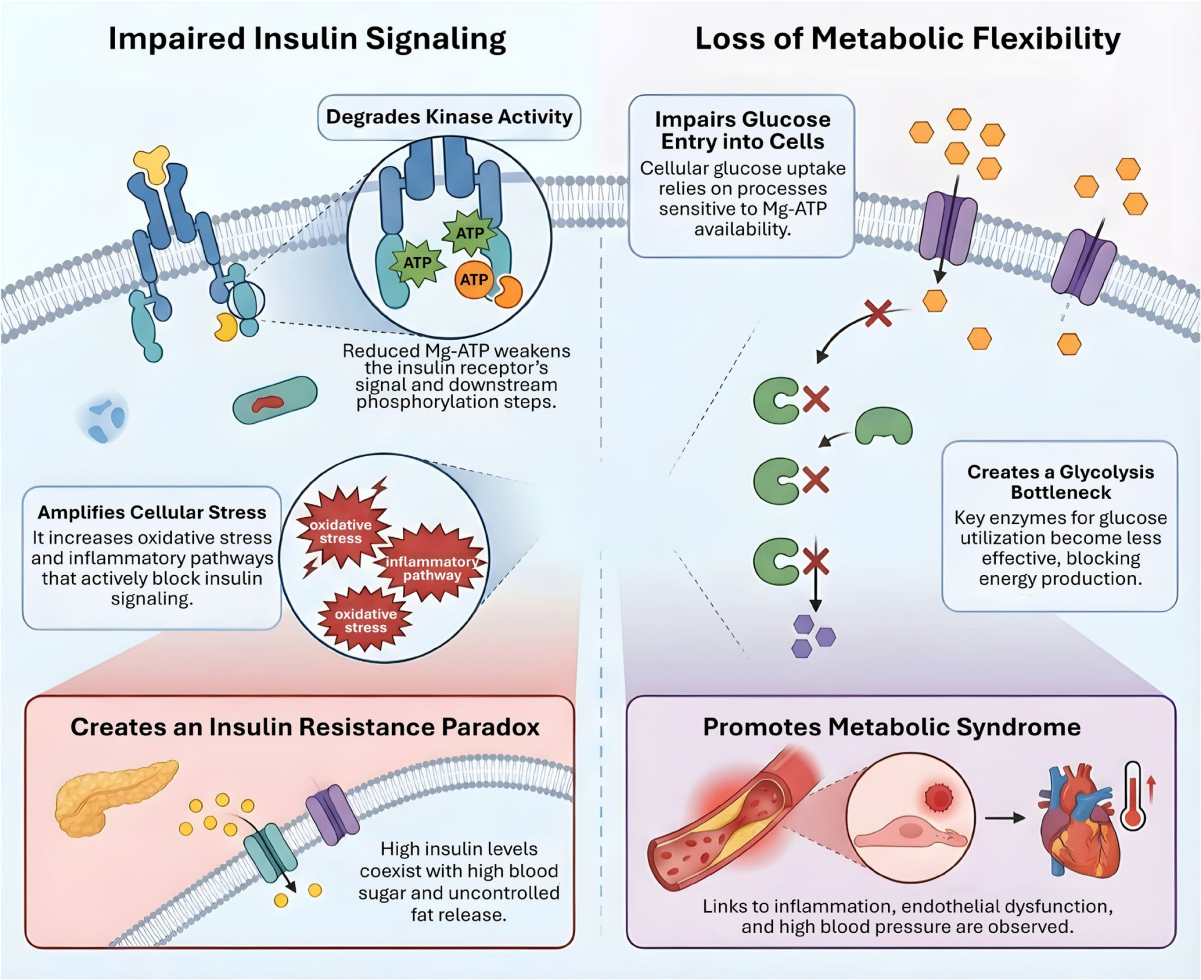
Intracellular magnesium deficiency links impaired insulin signaling to metabolic inflexibility. Reduced intracellular Mg^2+^ availability limits MgATP–dependent kinase activity downstream of the insulin receptor, weakening phosphorylation efficiency and blunting insulin signal propagation. Impaired signaling reduces GLUT4‐mediated glucose entry, while diminished MgATP availability further constrains glycolytic enzyme activity, creating a bottleneck in glucose utilization and energy production. In parallel, magnesium deficiency amplifies oxidative and inflammatory stress pathways that actively antagonize insulin signaling, reinforcing a state of insulin resistance despite elevated circulating insulin levels. Together, these processes promote loss of metabolic flexibility and establish a systemic insulin‐resistant state that predisposes to metabolic syndrome, vascular dysfunction, and cardiometabolic disease.

The earliest descriptions of magnesium deficient phenotypes emphasized impaired insulin action that could not be fully explained by changes in adiposity or caloric intake (Nadler et al. 1993; Reis et al. 2000; Resnick et al. 1988). Conceptually, Mg^2+^ scarcity degrades insulin signaling at two levels. First, it limits kinase chemistry. Tyrosine kinase activity of the insulin receptor and downstream phosphorylation steps require MgATP as the true substrate, so a decrease in free Mg^2+^ disproportionately reduces effective phosphoryl transfer even when total ATP appears preserved (Rosolova et al. 2000). The result is a shift from robust, switch‐like signal propagation to a leaky network in which IRS phosphorylation, PI3K activation, and AKT signaling are attenuated or temporally delayed, thereby uncoupling insulin binding from glucose transporter trafficking (Kearney et al. 2021). Second, Mg^2+^ deficiency amplifies counter‐regulatory signaling. Low intracellular Mg^2+^ has been associated with heightened oxidative stress, increased activation of stress kinases such as JNK and p38, and potentiation of inflammatory transcriptional programs (Fujita et al. 2023; Hotamisligil 2006). These stress pathways phosphorylate IRS proteins on inhibitory serine residues, further dampening insulin signaling in a manner that is difficult to rescue by insulin dose escalation alone (Jones et al. 2023). Importantly, this framework predicts a clinically familiar pattern: hyperinsulinaemia may coexist with persistent hepatic glucose output and defective skeletal muscle glucose disposal, while adipose tissue lipolysis remains insufficiently suppressed, feeding ectopic lipid deposition and reinforcing insulin resistance (Bkaily et al. 2025; Figure 5).

The metabolic syndrome is defined not by a single pathway lesion but by the loss of flexibility across tissues. Magnesium deficiency can contribute to this loss because it constrains multiple nodes required for rapid fuel switching. At the membrane interface, insulin stimulated glucose uptake depends on coordinated vesicle trafficking and cytoskeletal remodeling, processes that rely on ATP dependent kinases and phosphoinositide turnover, both sensitive to MgATP availability (Hosseini Dastgerdi et al. 2022; Oost et al. 2022). Within the cytosol, glycolytic enzymes and regulatory steps that depend on ATP, including hexokinase and phosphofructokinase control points, are indirectly affected when MgATP becomes limiting, creating a bottleneck at the very entry point of glucose utilization (Ichai et al. 2001; Liu, Wang, Wang, et al. 2025). In parallel, magnesium deficiency intersects with mitochondrial metabolism through altered redox balance and reactive oxygen species handling (Maier 2003; Zheltova et al. 2016). Excessive oxidative tone decreases insulin responsiveness and favors pro‐inflammatory macrophage polarization in adipose tissue, while endothelial dysfunction and sympathetic activation raise blood pressure, linking low Mg^2+^ to the full cardiometabolic cluster rather than glycaemia alone (Lopez‐Ridaura et al. 2004; Zheltova et al. 2016). These processes are consistent with cohort level observations that higher dietary magnesium intake is associated with lower incidence of type 2 diabetes and metabolic syndrome traits, including central adiposity, dyslipidaemia, and hypertension (Sales and Pedrosa Lde 2006; Veronese et al. 2016).

A central challenge in interpreting magnesium's role in metabolic syndrome is the complex directionality of its homeostasis, which we propose constitutes a self‐reinforcing “renal‐metabolic feedback loop.” Hyperglycaemia and hyperinsulinaemia directly alter renal tubular handling, while secondary factors such as diuretics and proton pump inhibitors further bias magnesium balance toward systemic loss (Gommers et al. 2022; Pham et al. 2007). In this context, the kidney functions not merely as a victim organ but as a “metabolic amplifier” of magnesium depletion. In insulin‐resistant states, glycosuria‐driven osmotic diuresis increases urinary magnesium excretion, while diabetic nephropathy further reduces reabsorptive reserve (Gommers et al. 2022; Sakaguchi et al. 2018). More critically, because insulin itself modulates epithelial magnesium transport, the onset of insulin resistance decouples hormonal cues from renal reabsorption capacity, effectively lowering the magnesium “ceiling” available to tissues even before the manifestation of overt kidney failure (Liu et al. 2020; Pham et al. 2012).

**FIGURE 6:**
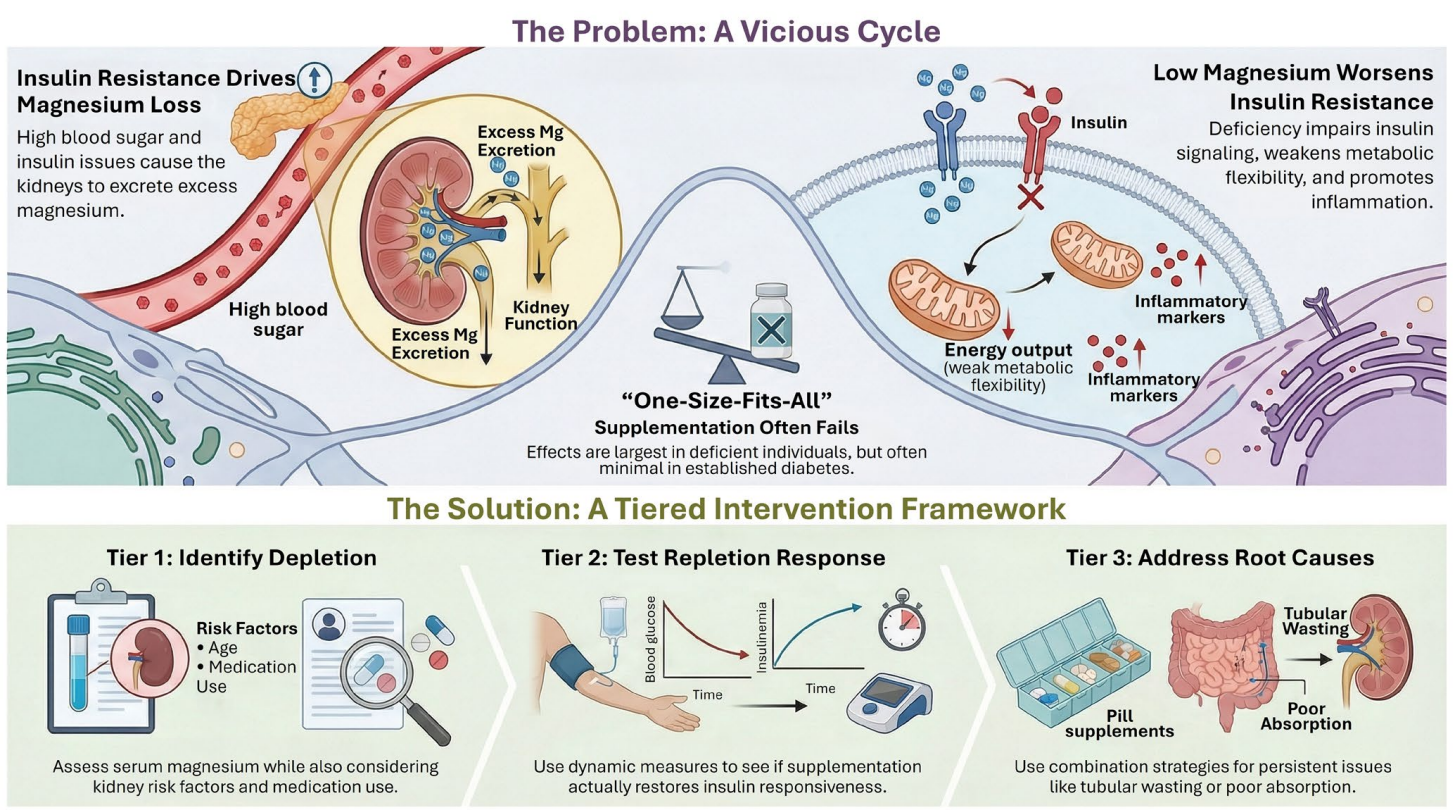
A bidirectional vicious cycle links magnesium loss to insulin resistance and defines a precision intervention strategy. Insulin resistance promotes renal magnesium wasting, while progressive Mg^2+^ loss impairs MgATP‐dependent insulin signaling, reduces metabolic flexibility, and enhances inflammatory stress, further exacerbating mitochondrial dysfunction and insulin resistance. A tiered intervention framework is proposed, comprising identification of magnesium depletion, functional testing of repletion response, and correction of persistent drivers such as renal magnesium wasting or impaired absorption. Together, this schematic highlights magnesium deficiency as a modifiable driver of insulin resistance that requires precision, mechanism‐informed intervention.

This physiology has direct implications for trials. Meta analyses of randomized studies generally support modest improvements in fasting glucose, insulin, and HOMA‐IR with magnesium supplementation, but effect sizes are variable and appear largest in individuals with baseline hypomagnesemia or impaired glucose regulation rather than in unselected populations (Drenthen et al. 2024; Simental‐Mendia et al. 2016). In prediabetes, targeted repletion has been reported to improve glycaemic indices and insulin sensitivity surrogates, consistent with the idea that restoring MgATP coupling can unmask latent insulin responsiveness (Drenthen et al. 2024; Guerrero‐Romero et al. 2015). In established diabetes, however, supplementation does not uniformly improve insulin sensitivity, especially when insulin resistance is driven by advanced ectopic lipid burden, inflammation, or comorbid kidney disease, underscoring the need for mechanistically informed stratification rather than one size dosing (Drenthen et al. 2024; Maqrashi et al. 2025; Figure 6).

A translationally useful model is therefore a tiered framework. Tier 1 identifies magnesium depletion, using serum magnesium while recognizing its limitations as a proxy for intracellular pools, and integrates renal risk factors and medication exposures (Gommers et al. 2022; Pham et al. 2007; Pitliya et al. 2024). Tier 2 tests whether repletion restores signaling responsiveness, assessed by dynamic measures such as postprandial glycaemia, clamp‐derived indices where feasible, or validated surrogate panels (Drenthen et al. 2024; Guerrero‐Romero et al. 2015). Tier 3 addresses persistence mechanisms, including tubular magnesium wasting, inflammatory stress, and mitochondrial dysfunction, which may require combination strategies beyond simple oral supplementation, such as correcting contributing medications, addressing gastrointestinal absorption, or developing targeted delivery approaches that better restore intracellular Mg^2+^ in insulin‐responsive tissues (Gommers et al. 2022; Pham et al. 2007). In sum, intracellular magnesium deficiency provides a mechanistic bridge between molecular bioenergetics and the clinical syndrome of insulin resistance. It can weaken insulin signal transduction through MgATP‐constrained phosphorylation, impair glycolytic entry and metabolic flexibility, and amplify stress kinase and inflammatory circuits that entrench the metabolic syndrome state. The clinical corollary is not that magnesium is a universal therapy, but that it is a rational, testable modifier of insulin resistance when deficiency is present and when renal and inflammatory drivers are simultaneously addressed.

## 5 The Aging Connection: Mitochondrial Magnesium in Senescence

**FIGURE 7:**
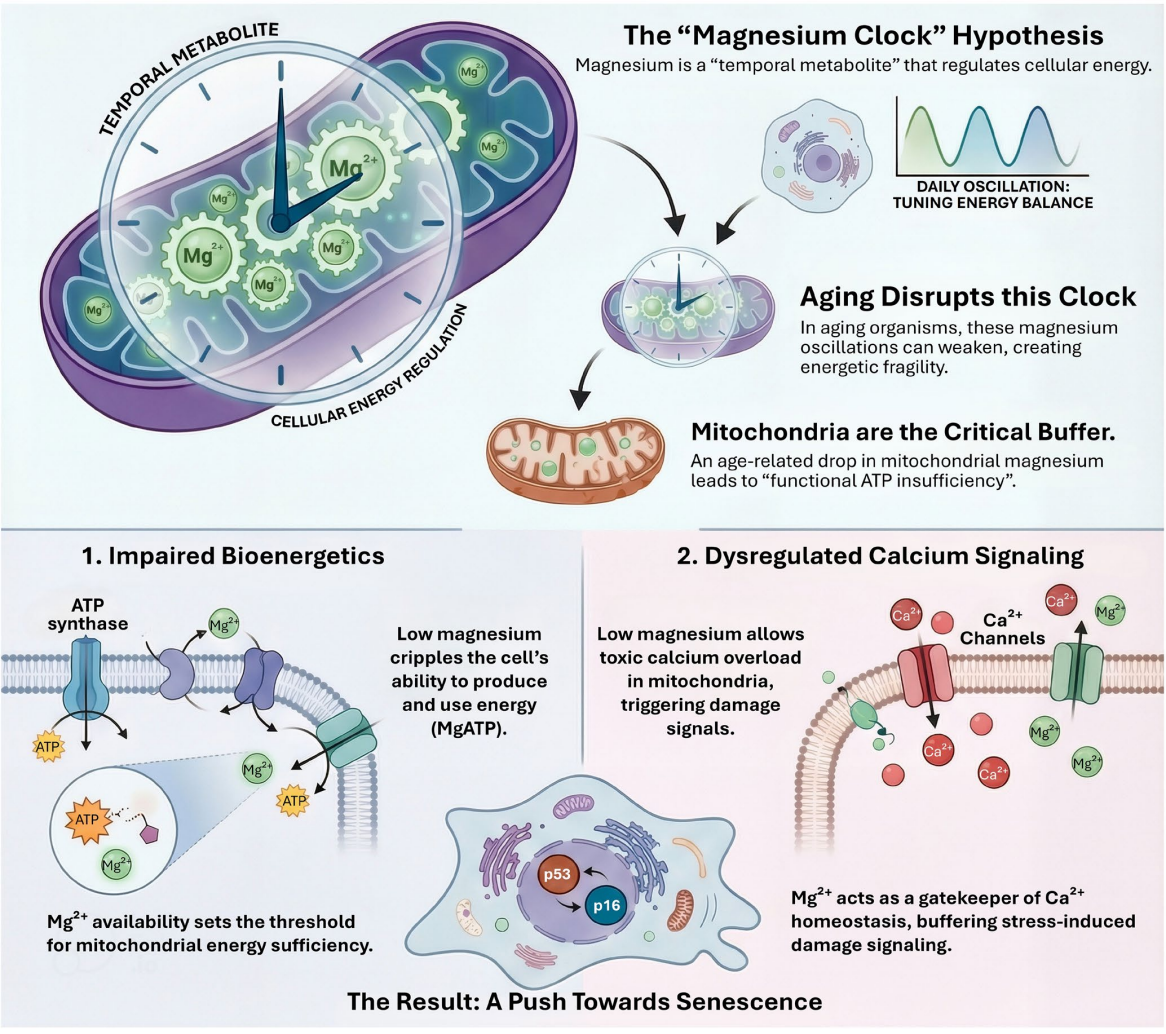
The “magnesium clock” hypothesis links mitochondrial magnesium dynamics to aging and senescence. This schematic proposes that magnesium functions as a temporal metabolite that tunes cellular energy balance through oscillatory regulation of mitochondrial Mg^2+^ availability. Under physiological conditions, rhythmic magnesium dynamics support Mg–ATP production and maintain alignment between energy demand and mitochondrial capacity. With aging, these magnesium oscillations weaken, creating energetic fragility. Mitochondria act as the critical buffering compartment, and age‐associated decline in mitochondrial Mg^2+^ leads to a state of functional ATP insufficiency despite preserved organelle mass.

**FIGURE 8:**
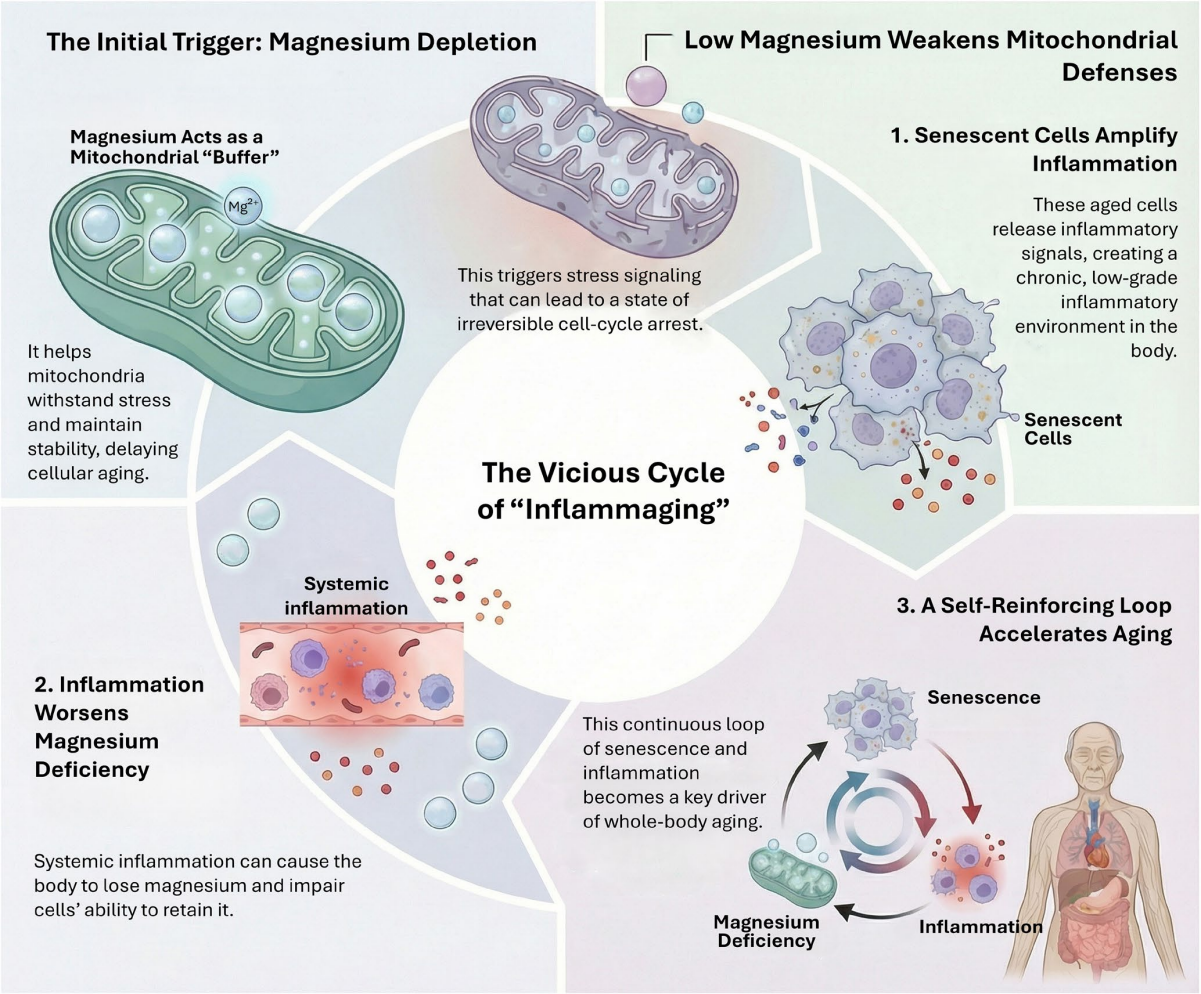
Magnesium depletion initiates a self‐reinforcing cycle of inflammaging and organismal aging. Under physiological conditions, mitochondrial Mg^2+^ buffers bioenergetic stress and supports cellular resilience. Decline in intracellular and mitochondrial magnesium weakens mitochondrial defenses, promotes cellular senescence, and amplifies inflammatory signaling, which in turn accelerates further magnesium loss. This reciprocal interaction generates a self‐reinforcing loop in which magnesium deficiency, mitochondrial dysfunction, and inflammation mutually amplify one another, progressively accelerating tissue dysfunction and organismal aging. Together, the model positions magnesium depletion not merely as a consequence of aging, but as an active driver of inflammaging that links mitochondrial failure to systemic decline.

Aging is characterized by an increasing frequency of bioenergetic “near misses” that eventually lead to irreversible cellular senescence. This trajectory is fundamentally linked to the metabolic rigidities discussed in Section 4, as the same MgATP‐constrained failures that drive insulin resistance also narrow the margin for mitochondrial stress tolerance in the aging organism. The “Magnesium Clock” hypothesis suggests that age‐dependent drift in mitochondrial magnesium acts as a temporal regulator that biases cells toward growth arrest by narrowing the margin between energetic demand and organelle tolerance (Figure 7). Within the mitochondrial matrix, the MRS2‐mediated magnesium set point serves as a “cationic rheostat” that restrains MCU‐mediated calcium uptake. When mitochondrial magnesium is lost, the resulting calcium‐triggered mitochondrial catastrophe accelerates the engagement of p53 and p16 pathways, stabilizing senescence‐associated growth arrest. While this model provides a strong conceptual synthesis, it is essential to distinguish its mechanistic plausibility from demonstrated lifespan causality. While magnesium restriction is known to accelerate senescence in vitro, direct longitudinal evidence tracking mitochondrial magnesium dynamics across the natural aging process of an organism remains limited. Nevertheless, this process is further amplified at the organismal level by “inflammaging,” where inflammatory signaling promotes renal magnesium wasting and senescent cells amplify the inflammatory milieu, creating a self‐reinforcing loop of systemic decline (Figure 8).

### 5.1 The Magnesium Clock and Age‐Related Bioenergetic Decline

The “Magnesium Clock” hypothesis originates from the observation that cytosolic magnesium is not static across the day. Instead, magnesium levels oscillate in a circadian manner and, by modulating global phosphorylation potential, can gate core timekeeping processes and energy balance (Feeney et al. 2016). This finding reframes magnesium as a temporal metabolite: it does not merely support enzymes; it periodically rewrites what ATP can do in vivo by tuning MgATP availability and kinase throughput. In aging organisms, circadian rhythms dampen and fragment, and mitochondrial output becomes noisier. A plausible extension is that magnesium oscillations also lose amplitude or become desynchronised across compartments, creating phases in which the cell's phosphorylation economy is intrinsically fragile. The key unresolved question is compartment specificity. If mitochondrial magnesium is the dominant buffer that stabilizes MgATP generation during fluctuating demand, then age‐related changes in mitochondrial membrane potential or transporter regulation could translate circadian misalignment into repeated episodes of “functional ATP insufficiency,” even when bulk ATP appears preserved. This sub‐aim therefore motivates a mechanistic map linking timekeeping, mitochondrial ion handling, and the progressive narrowing of bioenergetic resilience that precedes senescence.

### 5.2 Magnesium Homeostasis as a Driver of Cellular Aging

Direct experimental evidence supports a causal role for magnesium deficiency in driving senescence‐like states. Magnesium restriction accelerates senescence in cultured human fibroblasts, consistent with the idea that inadequate magnesium is not a passive correlate of aging but an active modifier of fate (Killilea and Ames 2008). Similarly, perturbing magnesium entry pathways can be sufficient to trigger senescence programmes: targeted silencing of TRPM7, a major magnesium‐permeable chanzyme, induces replicative senescence in proliferative cancer cells (Yee et al. 2012). These data converge on a unifying principle: when magnesium supply is constrained, the cell can retain mitochondria yet lose the ability to sustain phosphorylation‐dependent homeostasis under stress, making growth arrest and secretory remodeling more likely outcomes. How does the mitochondrion enter this logic? Mitochondrial magnesium homeostasis is increasingly understood as a regulated variable that controls energetic vulnerability. Work in mammalian systems indicates that mitochondrial magnesium balance can determine cellular energy metabolism and stress susceptibility (Yamanaka et al. 2016). At the molecular level, the mitochondrial magnesium channel MRS2 provides the best‐defined portal for matrix magnesium control, and recent structural biology has transformed this topic from inference to mechanism. Multiple high‐resolution structures show that MRS2 forms a gated channel whose conformational states can be captured in closed and open configurations, revealing principles of selectivity and regulation that were previously assigned by analogy to bacterial CorA transporters (Lai, Balaraman, et al. 2023; Li, Liu, Wallerstein, et al. 2025; Uthayabalan et al. 2023). In parallel, functional studies position MRS2 as a node that links mitochondrial magnesium availability to broader metabolic programmes, including developmental metabolic programming (Ponnusamy et al. 2024) and feedback regulation through defined protein domains (Mastrototaro et al. 2016). Together, these studies suggest that matrix magnesium is not merely “stored” but actively governed, making it realistic to propose that aging could shift the set point of mitochondrial magnesium through altered gating, expression, or membrane potential, and thereby reshape the probability landscape for senescence initiation.

A second mechanistic bridge from mitochondrial magnesium to senescence is calcium control. Mitochondrial calcium handling is now recognized as a key determinant of whether cells enter senescence or proceed to death programmes, particularly under chronic stress (Wiley et al. 2016). Strikingly, mitochondrial magnesium has been proposed as a “cationic rheostat” that restrains MCU‐mediated calcium uptake (Gallage and Gil 2016), and reduced matrix magnesium is associated with increased calcium influx and permeability transition pore susceptibility in this framework. Complementary work further supports the concept that MRS2‐mediated magnesium uptake is required to prevent mitochondrial calcium overload and preserve viability (Herranz and Gil 2018). These findings place mitochondrial magnesium at a strategic interface: it simultaneously supports MgATP‐dependent bioenergetics and limits calcium‐triggered mitochondrial catastrophe. Senescence, in this view, can emerge when magnesium loss compresses both margins at once, lowering ATP‐linked repair capacity while increasing calcium‐linked damage signaling, thereby accelerating the engagement of p53 and p16 pathways and stabilizing arrest.

### 5.3 Mitochondrial Magnesium in Senescence and Systemic Aging

Cellular senescence is not only a cell‐autonomous endpoint; it is a tissue‐level amplifier of aging through SASP‐driven inflammation, fibrosis, and metabolic rewiring. Mitochondrial dysfunction can induce a distinctive senescence state (MiDAS) with a specialized secretory output (Yarbro et al. 2020), and conceptual syntheses emphasize that senescence is heterogeneous and mechanistically plural, with metabolism functioning as both trigger and maintenance system (Margand et al. 2025; Wiley and Campisi 2021). Magnesium fits naturally into this pluralism as a modulator that can bias which senescence routes dominate in a given tissue: magnesium‐limited contexts may favor mitochondrial stress signaling and altered NAD and AMPK tone, whereas magnesium‐replete contexts may buffer transient mitochondrial insults and delay the consolidation of arrest. At the organismal level, associations between magnesium status and aging‐linked traits provide circumstantial but suggestive support for this model. Dietary and total magnesium intake has been analyzed in relation to frailty risk in older women (Struijk et al. 2024), consistent with magnesium availability tracking functional reserve at the whole‐body scale. In addition, recent work links magnesium‐related nutritional patterns to telomere dynamics in older adults (Dhillon et al. 2023), connecting magnesium to a canonical aging biomarker that integrates oxidative stress and replicative history.

While such studies align with a mechanistic narrative in which chronic, mild magnesium insufficiency increases the cumulative probability of senescence entry by weakening mitochondrial performance over time, they cannot yet resolve the direction of causality at the organismal level. It is essential to distinguish the mechanistic plausibility of this model from demonstrated lifespan causality. While the link between magnesium deficiency and cellular senescence is robustly supported in vitro, direct longitudinal evidence tracking mitochondrial magnesium dynamics across the natural aging process of a whole organism remains limited. Finally, inflammaging creates a feedback environment in which magnesium and senescence can reinforce each other. Aging is characterized by sterile, systemic inflammation, and macrophage immunometabolism has been highlighted as a critical axis linking mitochondrial dysfunction, senescence, CD38 activity, and NAD decline (Ponnusamy et al. 2024). Inflammatory signaling can promote renal magnesium wasting and impair cellular magnesium retention, while senescent cells amplify inflammatory tone, creating a loop that is plausibly magnesium‐sensitive (Ghosh‐Choudhary et al. 2021). Consequently, while we position mitochondrial magnesium as a “buffer of buffers” that stabilizes ATP‐linked repair and calcium‐linked tolerance, this framework should be viewed as a theoretical synthesis that necessitates future longitudinal studies to confirm its role as a primary driver of organismal aging. The implication is that mitochondrial magnesium may act as a “buffer of buffers,” stabilizing both ATP‐linked repair and calcium‐linked tolerance, thereby delaying not only senescence onset but also the inflammatory milieu that sustains senescence burden. As summarized in Figure 8, magnesium depletion initiates a self‐reinforcing cycle linking mitochondrial fragility, senescence‐associated inflammation, and systemic aging. In summary, the aging connection is not simply that magnesium “supports mitochondria,” but that mitochondrial magnesium may encode a time‐dependent constraint on metabolic fidelity. The circadian magnesium clock provides a conceptual entry point (Feeney et al. 2016), MRS2 biology supplies molecular levers, and calcium rheostat models define a catastrophe‐prevention mechanism (Lai, Balaraman, et al. 2023; Li, Liu, Wallerstein, et al. 2025; Mastrototaro et al. 2016; Ponnusamy et al. 2024; Uthayabalan et al. 2023). Translationally, this motivates a sharper distinction between serum magnesium and compartmental magnesium, and argues for aging research to incorporate mitochondrial magnesium state as both a biomarker and a candidate driver of senescence dynamics.

## 6 Therapeutic Perspectives: Restoring the Bioenergetic Checkpoint

At the organellar level, the mitochondrial magnesium channel MRS2 offers a deeper opportunity for precision intervention. Moving forward, a particularly exciting direction lies in longitudinal in vivo studies that track compartment‐specific mitochondrial Mg^2+^ dynamics across the natural lifespan. Such studies are required to move beyond hypothesis‐generating synthesis and provide definitive evidence for the role of magnesium drift in organismal aging. Future research should prioritize testing whether the genetic or pharmacological preservation of MRS2‐dependent matrix magnesium is sufficient to delay the onset of frailty or reduce the cumulative senescence burden in aging models. Integrating renal handling, medication exposure, disease stage, and mechanistic biomarkers should define therapeutic windows in which restoring Mg^2+^ homeostasis can meaningfully preserve cellular energetic integrity. In this framework, restoring Mg^2+^ homeostasis—anchored in the robust principles of transporter biology and MgATP‐centered energetics—becomes a targeted metabolic intervention to preserve cellular integrity and healthspan.

## Author Contributions

Y.‐J.H. and C.‐J.L. conceived the overall concept of the study, while Y.‐J.H., C.‐W.H., K.‐H.T., and C.‐Y.W. contributed to the development of the original idea. C.‐W.H., A.P.T., B.W., and C.‐Y.W. prepared the manuscript draft. Y.‐J.H. and C.‐J.L. participated in the review and revision of the manuscript. All authors discussed the results and contributed to the final version of the manuscript.

## Funding

This research was funded by the National Science and Technology Council (114‐2628‐B075B‐001‐MY3) and Kaohsiung Veterans General Hospital (KSVGH‐115‐056, KSVGH‐115‐061).

## Conflicts of Interest

The authors declare no conflicts of interest.

## Data Availability

Data sharing not applicable to this article as no datasets were generated or analysed during the current study.

## References

[acel70578-bib-0001] Anselme, M. , H. He , C. Lai , W. Luo , and S. Zhong . 2025. “Targeting Mitochondrial Transporters and Metabolic Reprogramming for Disease Treatment.” Journal of Translational Medicine 23, no. 1: 1111. 10.1186/s12967-025-06976-4. 41102706 PMC12532930

[acel70578-bib-0002] Barbagallo, M. , and L. J. Dominguez . 2007. “Magnesium Metabolism in Type 2 Diabetes Mellitus, Metabolic Syndrome and Insulin Resistance.” Archives of Biochemistry and Biophysics 458, no. 1: 40–47. 10.1016/j.abb.2006.05.007. 16808892

[acel70578-bib-0003] Bernardi, P. , and F. Di Lisa . 2015. “The Mitochondrial Permeability Transition Pore: Molecular Nature and Role as a Target in Cardioprotection.” Journal of Molecular and Cellular Cardiology 78: 100–106. 10.1016/j.yjmcc.2014.09.023. 25268651 PMC4294587

[acel70578-bib-0004] Bhargava, P. , and R. G. Schnellmann . 2017. “Mitochondrial Energetics in the Kidney.” Nature Reviews. Nephrology 13, no. 10: 629–646. 10.1038/nrneph.2017.107. 28804120 PMC5965678

[acel70578-bib-0005] Bkaily, G. , A. Jazzar , A. Abou‐Aichi , and D. Jacques . 2025. “Pathophysiology of Prediabetes Hyperinsulinemia and Insulin Resistance in the Cardiovascular System.” Biomedicine 13, no. 8: 1842. 10.3390/biomedicines13081842. PMC12383672 40868096

[acel70578-bib-0006] Chen, Y. S. , G. Kozlov , R. Fakih , et al. 2020. “Mg(2+)‐ATP Sensing in CNNM, a Putative Magnesium Transporter.” Structure 28, no. 3: 324–335.e324. 10.1016/j.str.2019.11.016. 31864811

[acel70578-bib-0007] Christov, M. , S. S. Waikar , R. C. Pereira , et al. 2013. “Plasma FGF23 Levels Increase Rapidly After Acute Kidney Injury.” Kidney International 84, no. 4: 776–785. 10.1038/ki.2013.150. 23657144 PMC3766419

[acel70578-bib-0008] Chubanov, V. , S. Ferioli , A. Wisnowsky , et al. 2016. “Epithelial Magnesium Transport by TRPM6 Is Essential for Prenatal Development and Adult Survival.” eLife 5: 20914. 10.7554/eLife.20914. PMC5218537 27991852

[acel70578-bib-0009] Daw, C. C. , K. Ramachandran , B. T. Enslow , et al. 2020. “Lactate Elicits ER‐Mitochondrial Mg(2+) Dynamics to Integrate Cellular Metabolism.” Cell 183, no. 2: 474–489.e417. 10.1016/j.cell.2020.08.049. 33035451 PMC7572828

[acel70578-bib-0010] de Baaij, J. H. , J. G. Hoenderop , and R. J. Bindels . 2015. “Magnesium in Man: Implications for Health and Disease.” Physiological Reviews 95, no. 1: 1–46. 10.1152/physrev.00012.2014. 25540137

[acel70578-bib-0011] Dhillon, V. S. , P. Deo , P. Thomas , and M. Fenech . 2023. “Low Magnesium in Conjunction With High Homocysteine and Less Sleep Accelerates Telomere Attrition in Healthy Elderly Australian.” International Journal of Molecular Sciences 24, no. 2: 982. 10.3390/ijms24020982. 36674498 PMC9866301

[acel70578-bib-0012] Dimke, H. , J. G. Hoenderop , and R. J. Bindels . 2011. “Molecular Basis of Epithelial Ca2+ and Mg2+ Transport: Insights From the TRP Channel Family.” Journal of Physiology 589, no. Pt 7: 1535–1542. 10.1113/jphysiol.2010.199869. 21041532 PMC3099013

[acel70578-bib-0013] Drenthen, L. C. A. , J. H. F. de Baaij , L. Rodwell , A. E. van Herwaarden , C. J. Tack , and B. E. de Galan . 2024. “Oral Magnesium Supplementation Does Not Affect Insulin Sensitivity in People With Insulin‐Treated Type 2 Diabetes and a Low Serum Magnesium: A Randomised Controlled Trial.” Diabetologia 67, no. 1: 52–61. 10.1007/s00125-023-06029-9. 37922013 PMC10709477

[acel70578-bib-0014] Faubert, B. , A. Solmonson , and R. J. DeBerardinis . 2020. “Metabolic Reprogramming and Cancer Progression.” Science 368, no. 6487: 5473. 10.1126/science.aaw5473. PMC7227780 32273439

[acel70578-bib-0015] Feeney, K. A. , L. L. Hansen , M. Putker , et al. 2016. “Daily Magnesium Fluxes Regulate Cellular Timekeeping and Energy Balance.” Nature 532, no. 7599: 375–379. 10.1038/nature17407. 27074515 PMC4886825

[acel70578-bib-0016] Fujita, K. , Y. Shindo , Y. Katsuta , M. Goto , K. Hotta , and K. Oka . 2023. “Intracellular Mg(2+) Protects Mitochondria From Oxidative Stress in Human Keratinocytes.” Communications Biology 6, no. 1: 868. 10.1038/s42003-023-05247-6. 37620401 PMC10449934

[acel70578-bib-0017] Funato, Y. , and H. Miki . 2022. “The Emerging Roles and Therapeutic Potential of Cyclin M/CorC Family of Mg(2+) Transporters.” Journal of Pharmacological Sciences 148, no. 1: 14–18. 10.1016/j.jphs.2021.09.004. 34924118

[acel70578-bib-0018] Gallage, S. , and J. Gil . 2016. “Mitochondrial Dysfunction Meets Senescence.” Trends in Biochemical Sciences 41, no. 3: 207–209. 10.1016/j.tibs.2016.01.005. 26874922

[acel70578-bib-0019] Ghosh‐Choudhary, S. K. , J. Liu , and T. Finkel . 2021. “The Role of Mitochondria in Cellular Senescence.” FASEB Journal 35, no. 12: e21991. 10.1096/fj.202101462R. 34758157 PMC8720272

[acel70578-bib-0020] Gimenez‐Mascarell, P. , I. Oyenarte , I. Gonzalez‐Recio , et al. 2019. “Structural Insights Into the Intracellular Region of the Human Magnesium Transport Mediator CNNM4.” International Journal of Molecular Sciences 20, no. 24: 6279. 10.3390/ijms20246279. 31842432 PMC6940986

[acel70578-bib-0021] Gimenez‐Mascarell, P. , I. Oyenarte , S. Hardy , et al. 2017. “Structural Basis of the Oncogenic Interaction of Phosphatase PRL‐1 With the Magnesium Transporter CNNM2.” Journal of Biological Chemistry 292, no. 3: 786–801. 10.1074/jbc.M116.759944. 27899452 PMC5247653

[acel70578-bib-0022] Gommers, L. M. M. , J. G. J. Hoenderop , and J. H. F. de Baaij . 2022. “Mechanisms of Proton Pump Inhibitor‐Induced Hypomagnesemia.” Acta Physiologica (Oxford, England) 235, no. 4: e13846. 10.1111/apha.13846. 35652564 PMC9539870

[acel70578-bib-0023] Guerrero‐Romero, F. , L. E. Simental‐Mendia , G. Hernandez‐Ronquillo , and M. Rodriguez‐Moran . 2015. “Oral Magnesium Supplementation Improves Glycaemic Status in Subjects With Prediabetes and Hypomagnesaemia: A Double‐Blind Placebo‐Controlled Randomized Trial.” Diabetes & Metabolism 41, no. 3: 202–207. 10.1016/j.diabet.2015.03.010. 25937055

[acel70578-bib-0024] Gulerez, I. , Y. Funato , H. Wu , et al. 2016. “Phosphocysteine in the PRL‐CNNM Pathway Mediates Magnesium Homeostasis.” EMBO Reports 17, no. 12: 1890–1900. 10.15252/embr.201643393. 27856537 PMC5283600

[acel70578-bib-0025] Guo, H. , and J. L. Rubinstein . 2022. “Structure of ATP Synthase Under Strain During Catalysis.” Nature Communications 13, no. 1: 2232. 10.1038/s41467-022-29893-2. PMC9038767 35468906

[acel70578-bib-0026] Gupta, R. K. , and R. D. Moore . 1980. “31P NMR Studies of Intracellular Free Mg2+ in Intact Frog Skeletal Muscle.” Journal of Biological Chemistry 255, no. 9: 3987–3993. 6966281

[acel70578-bib-0027] Gupta, R. K. , and W. D. Yushok . 1980. “Noninvasive 31P NMR Probes of Free Mg2+, MgATP, and MgADP in Intact Ehrlich Ascites Tumor Cells.” Proceedings of the National Academy of Sciences of the United States of America 77, no. 5: 2487–2491. 10.1073/pnas.77.5.2487. 6930646 PMC349425

[acel70578-bib-0028] He, Z. , Y. C. Tu , C. W. Tsai , et al. 2025. “Structure and Function of the Human Mitochondrial MRS2 Channel.” Nature Structural & Molecular Biology 32, no. 3: 459–468. 10.1038/s41594-024-01420-5. PMC11922672 39609651

[acel70578-bib-0029] Herranz, N. , and J. Gil . 2018. “Mechanisms and Functions of Cellular Senescence.” Journal of Clinical Investigation 128, no. 4: 1238–1246. 10.1172/JCI95148. 29608137 PMC5873888

[acel70578-bib-0030] Hosseini Dastgerdi, A. , M. Ghanbari Rad , and N. Soltani . 2022. “The Therapeutic Effects of Magnesium in Insulin Secretion and Insulin Resistance.” Advanced Biomedical Research 11: 54. 10.4103/abr.abr_366_21. 35982863 PMC9379913

[acel70578-bib-0031] Hotamisligil, G. S. 2006. “Inflammation and Metabolic Disorders.” Nature 444, no. 7121: 860–867. 10.1038/nature05485. 17167474

[acel70578-bib-0032] Ichai, C. , M. Y. El‐Mir , V. Nogueira , et al. 2001. “Exogenous Mg‐ATP Induces a Large Inhibition of Pyruvate Kinase in Intact Rat Hepatocytes.” Journal of Biological Chemistry 276, no. 9: 6398–6403. 10.1074/jbc.M004169200. 11104754

[acel70578-bib-0033] Jones, I. C. , R. Carnagarin , J. Armstrong , et al. 2023. “Pigment Epithelium‐Derived Factor: Inhibition of Phosphorylation of Insulin Receptor (IR)/IR Substrate (IRS), Osteogeneration From Adipocytes, and Increased Levels due to Doxorubicin Exposure.” Pharmaceutics 15, no. 7: 1960. 10.3390/pharmaceutics15071960. 37514146 PMC10384968

[acel70578-bib-0035] Kang, H. M. , S. H. Ahn , P. Choi , et al. 2015. “Defective Fatty Acid Oxidation in Renal Tubular Epithelial Cells Has a Key Role in Kidney Fibrosis Development.” Nature Medicine 21, no. 1: 37–46. 10.1038/nm.3762. PMC4444078 25419705

[acel70578-bib-0036] Kearney, A. L. , D. M. Norris , M. Ghomlaghi , et al. 2021. “Akt Phosphorylates Insulin Receptor Substrate to Limit PI3K‐Mediated PIP3 Synthesis.” eLife 10: 66942. 10.7554/eLife.66942. PMC8277355 34253290

[acel70578-bib-0037] Khan, S. , and A. A. Khan . 2025. “Hypoparathyroidism: Diagnosis, Management and Emerging Therapies.” Nature Reviews. Endocrinology 21, no. 6: 360–374. 10.1038/s41574-024-01075-8. 39905273

[acel70578-bib-0038] Killilea, D. W. , and B. N. Ames . 2008. “Magnesium Deficiency Accelerates Cellular Senescence in Cultured Human Fibroblasts.” Proceedings of the National Academy of Sciences of the United States of America 105, no. 15: 5768–5773. 10.1073/pnas.0712401105. 18391207 PMC2311331

[acel70578-bib-0039] Kirichok, Y. , G. Krapivinsky , and D. E. Clapham . 2004. “The Mitochondrial Calcium Uniporter Is a Highly Selective Ion Channel.” Nature 427, no. 6972: 360–364. 10.1038/nature02246. 14737170

[acel70578-bib-0040] Kleczkowski, L. A. , and A. U. Igamberdiev . 2023. “Magnesium and Cell Energetics: At the Junction of Metabolism of Adenylate and Non‐Adenylate Nucleotides.” Journal of Plant Physiology 280: 153901. 10.1016/j.jplph.2022.153901. 36549033

[acel70578-bib-0041] Kleczkowski, L. A. , and A. U. Igamberdiev . 2025. “Adenylate‐Driven Equilibration of Both Ribo‐ and Deoxyribonucleotides Is Under Magnesium Control: Quantification of the Mg(2+)‐Signal.” Journal of Plant Physiology 304: 154380. 10.1016/j.jplph.2024.154380. 39709740

[acel70578-bib-0042] Lai, L. T. F. , J. Balaraman , F. Zhou , and D. Matthies . 2023. “Cryo‐EM Structures of Human Magnesium Channel MRS2 Reveal Gating and Regulatory Mechanisms.” Nature Communications 14, no. 1: 7207. 10.1038/s41467-023-42599-3. PMC10632456 37938562

[acel70578-bib-0043] Lai, Y. , Y. Zhang , S. Zhou , et al. 2023. “Structure of the Human ATP Synthase.” Molecular Cell 83, no. 12: 2137–2147.e2134. 10.1016/j.molcel.2023.04.029. 37244256

[acel70578-bib-0045] Li, M. , Y. Li , Y. Lu , et al. 2023. “Molecular Basis of Mg(2+) Permeation Through the Human Mitochondrial Mrs2 Channel.” Nature Communications 14, no. 1: 4713. 10.1038/s41467-023-40516-2. PMC10404273 37543649

[acel70578-bib-0046] Li, P. , S. Liu , J. Wallerstein , et al. 2025. “Closed and Open Structures of the Eukaryotic Magnesium Channel Mrs2 Reveal the Auto‐Ligand‐Gating Regulation Mechanism.” Nature Structural & Molecular Biology 32, no. 3: 491–501. 10.1038/s41594-024-01432-1. PMC11919701 39609652

[acel70578-bib-0047] Liamis, G. , E. Liberopoulos , F. Barkas , and M. Elisaf . 2014. “Diabetes Mellitus and Electrolyte Disorders.” World Journal of Clinical Cases 2, no. 10: 488–496. 10.12998/wjcc.v2.i10.488. 25325058 PMC4198400

[acel70578-bib-0048] Linkermann, A. , G. Chen , G. Dong , U. Kunzendorf , S. Krautwald , and Z. Dong . 2014. “Regulated Cell Death in AKI.” Journal of the American Society of Nephrology 25, no. 12: 2689–2701. 10.1681/ASN.2014030262. 24925726 PMC4243360

[acel70578-bib-0049] Liu, H. , N. Li , M. Jin , X. Miao , X. Zhang , and W. Zhong . 2020. “Magnesium Supplementation Enhances Insulin Sensitivity and Decreases Insulin Resistance in Diabetic Rats.” Iranian Journal of Basic Medical Sciences 23, no. 8: 990–998. 10.22038/ijbms.2020.40859.9650. 32952944 PMC7478262

[acel70578-bib-0050] Liu, H. , S. Wang , J. Wang , et al. 2025. “Energy Metabolism in Health and Diseases.” Signal Transduction and Targeted Therapy 10, no. 1: 69. 10.1038/s41392-025-02141-x. 39966374 PMC11836267

[acel70578-bib-0051] Liu, J. , Y. Wang , P. Qiao , et al. 2025. “Mechanisms of Cisplatin‐Induced Acute Kidney Injury: The Role of NRF2 in Mitochondrial Dysfunction and Metabolic Reprogramming.” Antioxidants (Basel) 14, no. 7: 775. 10.3390/antiox14070775. 40722880 PMC12291884

[acel70578-bib-0052] Lopez‐Ridaura, R. , W. C. Willett , E. B. Rimm , et al. 2004. “Magnesium Intake and Risk of Type 2 Diabetes in Men and Women.” Diabetes Care 27, no. 1: 134–140. 10.2337/diacare.27.1.134. 14693979

[acel70578-bib-0053] Madaris, T. R. , M. Venkatesan , S. Maity , et al. 2023. “Limiting Mrs2‐Dependent Mitochondrial Mg(2+) Uptake Induces Metabolic Programming in Prolonged Dietary Stress.” Cell Reports 42, no. 3: 112155. 10.1016/j.celrep.2023.112155. 36857182 PMC10134742

[acel70578-bib-0054] Maier, J. A. 2003. “Low Magnesium and Atherosclerosis: An Evidence‐Based Link.” Molecular Aspects of Medicine 24, no. 1–3: 137–146. 10.1016/s0098-2997(02)00095-x. 12537993

[acel70578-bib-0055] Maqrashi, N. A. , S. A. Busaidi , S. Al‐Rasbi , A. M. A. Alawi , and J. S. Al‐Maqbali . 2025. “Effect of Magnesium Supplements on Improving Glucose Control, Blood Pressure and Lipid Profile in Patients With Type 2 Diabetes Mellitus: A Systematic Review and Meta‐Analysis.” Sultan Qaboos University Medical Journal 25, no. 1: 382–394. 10.18295/2075-0528.2848. 40641714 PMC12244252

[acel70578-bib-0056] Margand, C. , P. Morgado‐Caceres , U. Ahumada‐Castro , J. C. Cardenas , N. Martin , and D. Bernard . 2025. “Emerging Role of Mitochondrial Calcium Levels in Cellular Senescence and in Switching Cell Fates.” Nat Aging 5, no. 7: 1177–1180. 10.1038/s43587-025-00887-1. 40419804

[acel70578-bib-0057] Mastrototaro, L. , A. Smorodchenko , J. R. Aschenbach , M. Kolisek , and G. Sponder . 2016. “Solute Carrier 41A3 Encodes for a Mitochondrial Mg(2+) Efflux System.” Scientific Reports 6: 27999. 10.1038/srep27999. 27302215 PMC4908428

[acel70578-bib-0058] Miguel, V. , J. Tituana , J. I. Herrero , et al. 2021. “Renal Tubule Cpt1a Overexpression Protects From Kidney Fibrosis by Restoring Mitochondrial Homeostasis.” Journal of Clinical Investigation 131, no. 5: e140695. 10.1172/JCI140695. 33465052 PMC7919728

[acel70578-bib-0059] Mittermeier, L. , L. Demirkhanyan , B. Stadlbauer , et al. 2019. “TRPM7 Is the Central Gatekeeper of Intestinal Mineral Absorption Essential for Postnatal Survival.” Proceedings of the National Academy of Sciences of the United States of America 116, no. 10: 4706–4715. 10.1073/pnas.1810633116. 30770447 PMC6410795

[acel70578-bib-0060] Nadler, J. L. , T. Buchanan , R. Natarajan , I. Antonipillai , R. Bergman , and R. Rude . 1993. “Magnesium Deficiency Produces Insulin Resistance and Increased Thromboxane Synthesis.” Hypertension 21, no. 6 Pt 2: 1024–1029. 10.1161/01.hyp.21.6.1024. 8505087

[acel70578-bib-0061] Nam, K. , A. R. A. Thodika , S. Tischlik , et al. 2024. “Magnesium Induced Structural Reorganization in the Active Site of Adenylate Kinase.” Science Advances 10, no. 32: eado5504. 10.1126/sciadv.ado5504. 39121211 PMC11313852

[acel70578-bib-0062] O'Connor, P. M. 2006. “Renal Oxygen Delivery: Matching Delivery to Metabolic Demand.” Clinical and Experimental Pharmacology & Physiology 33, no. 10: 961–967. 10.1111/j.1440-1681.2006.04475.x. 17002675

[acel70578-bib-0063] Oost, L. J. , S. Kurstjens , C. Ma , J. G. J. Hoenderop , C. J. Tack , and J. H. F. de Baaij . 2022. “Magnesium Increases Insulin‐Dependent Glucose Uptake in Adipocytes.” Frontiers in Endocrinology (Lausanne) 13: 986616. 10.3389/fendo.2022.986616. PMC9453642 36093068

[acel70578-bib-0064] Oost, L. J. , C. J. Tack , and J. H. F. de Baaij . 2023. “Hypomagnesemia and Cardiovascular Risk in Type 2 Diabetes.” Endocrine Reviews 44, no. 3: 357–378. 10.1210/endrev/bnac028. 36346820 PMC10166267

[acel70578-bib-0065] Osada, A. , M. Tanaka , Y. Sugiura , et al. 2025. “Altered Glycolipid Metabolism During Acute Kidney Injury Exacerbates Renal Inflammation.” Scientific Reports 16: 147. 10.1038/s41598-025-28897-4. 41331311 PMC12765023

[acel70578-bib-0066] Pham, P. C. , P. A. Pham , S. V. Pham , P. T. Pham , P. M. Pham , and P. T. Pham . 2014. “Hypomagnesemia: A Clinical Perspective.” International Journal of Nephrology and Renovascular Disease 7: 219–230. 10.2147/IJNRD.S42054. 24966690 PMC4062555

[acel70578-bib-0067] Pham, P. C. , P. M. Pham , and P. T. Pham . 2012. “Patients With Diabetes Mellitus Type 2 and Hypomagnesemia May Have Enhanced Glomerular Filtration via Hypocalcemia.” Clinical Nephrology 78, no. 6: 442–448. 10.5414/CN107525. 23073059

[acel70578-bib-0068] Pham, P. C. , P. M. Pham , S. V. Pham , J. M. Miller , and P. T. Pham . 2007. “Hypomagnesemia in Patients With Type 2 Diabetes.” Clinical Journal of the American Society of Nephrology 2, no. 2: 366–373. 10.2215/CJN.02960906. 17699436

[acel70578-bib-0069] Pilchova, I. , K. Klacanova , Z. Tatarkova , P. Kaplan , and P. Racay . 2017. “The Involvement of Mg(2+) in Regulation of Cellular and Mitochondrial Functions.” Oxidative Medicine and Cellular Longevity 2017: 6797460. 10.1155/2017/6797460. 28757913 PMC5516748

[acel70578-bib-0070] Pitliya, A. , S. S. Vasudevan , V. Batra , et al. 2024. “Global Prevalence of Hypomagnesemia in Type 2 Diabetes Mellitus ‐ a Comprehensive Systematic Review and Meta‐Analysis of Observational Studies.” Endocrine 84, no. 3: 842–851. 10.1007/s12020-023-03670-7. 38159172

[acel70578-bib-0072] Ponnusamy, T. , P. Velusamy , and S. Shanmughapriya . 2024. “Mrs2‐Mediated Mitochondrial Magnesium Uptake Is Essential for the Regulation of MCU‐Mediated Mitochondrial Ca^2+^ Uptake and Viability.” Mitochondrion 76: 101877. 10.1016/j.mito.2024.101877. 38599304 PMC12720483

[acel70578-bib-0073] Reis, M. A. , F. G. Reyes , M. J. Saad , and L. A. Velloso . 2000. “Magnesium Deficiency Modulates the Insulin Signaling Pathway in Liver but Not Muscle of Rats.” Journal of Nutrition 130, no. 2: 133–138. 10.1093/jn/130.2.133. 10720159

[acel70578-bib-0074] Resnick, L. M. , R. K. Gupta , H. Gruenspan , and J. H. Laragh . 1988. “Intracellular Free Magnesium in Hypertension: Relation to Peripheral Insulin Resistance.” Journal of Hypertension. Supplement 6, no. 4: S199–S201. 3241201

[acel70578-bib-0075] Rosolova, H. , O. Mayer Jr. , and G. M. Reaven . 2000. “Insulin‐Mediated Glucose Disposal Is Decreased in Normal Subjects With Relatively Low Plasma Magnesium Concentrations.” Metabolism 49, no. 3: 418–420. 10.1016/s0026-0495(00)90462-1. 10726923

[acel70578-bib-0076] Run, C. , Q. Yang , Z. Liu , B. OuYang , and J. J. Chou . 2015. “Molecular Basis of MgATP Selectivity of the Mitochondrial SCaMC Carrier.” Structure 23, no. 8: 1394–1403. 10.1016/j.str.2015.06.004. 26165595 PMC4526376

[acel70578-bib-0077] Sakaguchi, Y. , T. Hamano , and Y. Isaka . 2018. “Magnesium and Progression of Chronic Kidney Disease: Benefits Beyond Cardiovascular Protection?” Advances in Chronic Kidney Disease 25, no. 3: 274–280. 10.1053/j.ackd.2017.11.001. 29793667

[acel70578-bib-0078] Sales, C. H. , and F. Pedrosa Lde . 2006. “Magnesium and Diabetes Mellitus: Their Relation.” Clinical Nutrition 25, no. 4: 554–562. 10.1016/j.clnu.2006.03.003. 16690176

[acel70578-bib-0079] Schlingmann, K. P. , S. Weber , M. Peters , et al. 2002. “Hypomagnesemia With Secondary Hypocalcemia Is Caused by Mutations in TRPM6, a New Member of the TRPM Gene Family.” Nature Genetics 31, no. 2: 166–170. 10.1038/ng889. 12032568

[acel70578-bib-0080] Schmidt, E. , C. Narangoda , W. Norenberg , et al. 2022. “Structural Mechanism of TRPM7 Channel Regulation by Intracellular Magnesium.” Cellular and Molecular Life Sciences 79, no. 5: 225. 10.1007/s00018-022-04192-7. 35389104 PMC8989868

[acel70578-bib-0081] Simental‐Mendia, L. E. , A. Sahebkar , M. Rodriguez‐Moran , and F. Guerrero‐Romero . 2016. “A Systematic Review and Meta‐Analysis of Randomized Controlled Trials on the Effects of Magnesium Supplementation on Insulin Sensitivity and Glucose Control.” Pharmacological Research 111: 272–282. 10.1016/j.phrs.2016.06.019. 27329332

[acel70578-bib-0082] Sobti, M. , H. Ueno , H. Noji , and A. G. Stewart . 2021. “The Six Steps of the Complete F(1)‐ATPase Rotary Catalytic Cycle.” Nature Communications 12, no. 1: 4690. 10.1038/s41467-021-25029-0. PMC8333055 34344897

[acel70578-bib-0083] Soltoff, S. P. 1986. “ATP and the Regulation of Renal Cell Function.” Annual Review of Physiology 48: 9–31. 10.1146/annurev.ph.48.030186.000301. 3010834

[acel70578-bib-0084] Struijk, E. A. , T. T. Fung , H. A. Bischoff‐Ferrari , W. C. Willett , and E. Lopez‐Garcia . 2024. “Total Magnesium Intake and Risk of Frailty in Older Women.” Journal of Cachexia, Sarcopenia and Muscle 15, no. 4: 1275–1282. 10.1002/jcsm.13450. 38845194 PMC11294045

[acel70578-bib-0085] Sun, P. , Y. Liu , T. Ma , and J. Ding . 2020. “Structure and Allosteric Regulation of Human NAD‐Dependent Isocitrate Dehydrogenase.” Cell Discovery 6, no. 1: 94. 10.1038/s41421-020-00220-7. 33349631 PMC7752914

[acel70578-bib-0086] Traba, J. , A. Del Arco , M. R. Duchen , G. Szabadkai , and J. Satrustegui . 2012. “SCaMC‐1 Promotes Cancer Cell Survival by Desensitizing Mitochondrial Permeability Transition via ATP/ADP‐Mediated Matrix ca(2+) Buffering.” Cell Death and Differentiation 19, no. 4: 650–660. 10.1038/cdd.2011.139. 22015608 PMC3307981

[acel70578-bib-0087] Tran, M. , D. Tam , A. Bardia , et al. 2011. “PGC‐1alpha Promotes Recovery After Acute Kidney Injury During Systemic Inflammation in Mice.” Journal of Clinical Investigation 121, no. 10: 4003–4014. 10.1172/JCI58662. 21881206 PMC3195479

[acel70578-bib-0088] Uthayabalan, S. , N. Vishnu , M. Madesh , and P. B. Stathopulos . 2023. “The Human MRS2 Magnesium‐Binding Domain Is a Regulatory Feedback Switch for Channel Activity.” Life Science Alliance 6, no. 4: e202201742. 10.26508/lsa.202201742. 36754568 PMC9909464

[acel70578-bib-0089] Vargas‐Poussou, R. , F. Claverie‐Martin , C. Prot‐Bertoye , et al. 2023. “Possible Role for Rare TRPM7 Variants in Patients With Hypomagnesaemia With Secondary Hypocalcaemia.” Nephrology, Dialysis, Transplantation 38, no. 3: 679–690. 10.1093/ndt/gfac182. PMC9976740 35561741

[acel70578-bib-0090] Veronese, N. , S. Watutantrige‐Fernando , C. Luchini , et al. 2016. “Effect of Magnesium Supplementation on Glucose Metabolism in People With or at Risk of Diabetes: A Systematic Review and Meta‐Analysis of Double‐Blind Randomized Controlled Trials.” European Journal of Clinical Nutrition 70, no. 12: 1463. 10.1038/ejcn.2016.209. 27924111

[acel70578-bib-0091] Voets, T. , B. Nilius , S. Hoefs , et al. 2004. “TRPM6 Forms the Mg2+ Influx Channel Involved in Intestinal and Renal Mg2+ Absorption.” Journal of Biological Chemistry 279, no. 1: 19–25. 10.1074/jbc.M311201200. 14576148

[acel70578-bib-0092] Walder, R. Y. , D. Landau , P. Meyer , et al. 2002. “Mutation of TRPM6 Causes Familial Hypomagnesemia With Secondary Hypocalcemia.” Nature Genetics 31, no. 2: 171–174. 10.1038/ng901. 12032570

[acel70578-bib-0093] Weiner, I. D. , W. E. Mitch , and J. M. Sands . 2015. “Urea and Ammonia Metabolism and the Control of Renal Nitrogen Excretion.” Clinical Journal of the American Society of Nephrology 10, no. 8: 1444–1458. 10.2215/CJN.10311013. 25078422 PMC4527031

[acel70578-bib-0094] Wilde, B. R. , and H. R. Christofk . 2020. “O‐Mg! Lactate Drives Mg(2+) Mobilization.” Molecular Cell 80, no. 5: 762–763. 10.1016/j.molcel.2020.11.023. 33275887

[acel70578-bib-0095] Wiley, C. D. , and J. Campisi . 2021. “The Metabolic Roots of Senescence: Mechanisms and Opportunities for Intervention.” Nature Metabolism 3, no. 10: 1290–1301. 10.1038/s42255-021-00483-8. PMC8889622 34663974

[acel70578-bib-0096] Wiley, C. D. , M. C. Velarde , P. Lecot , et al. 2016. “Mitochondrial Dysfunction Induces Senescence With a Distinct Secretory Phenotype.” Cell Metabolism 23, no. 2: 303–314. 10.1016/j.cmet.2015.11.011. 26686024 PMC4749409

[acel70578-bib-0097] Yamanaka, R. , S. Tabata , Y. Shindo , et al. 2016. “Mitochondrial Mg(2+) Homeostasis Decides Cellular Energy Metabolism and Vulnerability to Stress.” Scientific Reports 6: 30027. 10.1038/srep30027. 27458051 PMC4960558

[acel70578-bib-0098] Yamazaki, D. , Y. Funato , J. Miura , et al. 2013. “Basolateral Mg2+ Extrusion via CNNM4 Mediates Transcellular Mg2+ Transport Across Epithelia: A Mouse Model.” PLoS Genetics 9, no. 12: e1003983. 10.1371/journal.pgen.1003983. 24339795 PMC3854942

[acel70578-bib-0099] Yarbro, J. R. , R. S. Emmons , and B. D. Pence . 2020. “Macrophage Immunometabolism and Inflammaging: Roles of Mitochondrial Dysfunction, Cellular Senescence, CD38, and NAD.” Immunometabolism 2, no. 3: e200026. 10.20900/immunometab20200026. 32774895 PMC7409778

[acel70578-bib-0100] Yee, N. S. , W. Zhou , M. Lee , and R. K. Yee . 2012. “Targeted Silencing of TRPM7 Ion Channel Induces Replicative Senescence and Produces Enhanced Cytotoxicity With Gemcitabine in Pancreatic Adenocarcinoma.” Cancer Letters 318, no. 1: 99–105. 10.1016/j.canlet.2011.12.007. 22166235 PMC3303226

[acel70578-bib-0101] Zhang, M. , Y. Ma , X. Ye , N. Zhang , L. Pan , and B. Wang . 2023. “TRP (Transient Receptor Potential) Ion Channel Family: Structures, Biological Functions and Therapeutic Interventions for Diseases.” Signal Transduction and Targeted Therapy 8, no. 1: 261. 10.1038/s41392-023-01464-x. 37402746 PMC10319900

[acel70578-bib-0102] Zheltova, A. A. , M. V. Kharitonova , I. N. Iezhitsa , and A. A. Spasov . 2016. “Magnesium Deficiency and Oxidative Stress: An Update.” Biomedicine (Taipei) 6, no. 4: 20. 10.7603/s40681-016-0020-6. 27854048 PMC5112180

[acel70578-bib-0103] Zhong, Y. , Y. Gao , D. Zhou , et al. 2022. “Structural Basis for the Activity and Regulation of Human Alpha‐Ketoglutarate Dehydrogenase Revealed by Cryo‐EM.” Biochemical and Biophysical Research Communications 602: 120–126. 10.1016/j.bbrc.2022.02.093. 35272141

